# Perovskite Nanocrystals Protected by Hermetically Sealing for Highly Bright and Stable Deep‐Blue Light‐Emitting Diodes

**DOI:** 10.1002/advs.202302906

**Published:** 2023-06-04

**Authors:** Yongju Hong, Chungman Yu, Hyeondoo Je, Jin Young Park, Taekyung Kim, Hionsuck Baik, Gracita M. Tomboc, Youngseo Kim, Jung Min Ha, Jinwhan Joo, Chai Won Kim, Han Young Woo, Sungnam Park, Dong Hoon Choi, Kwangyeol Lee

**Affiliations:** ^1^ Department of Chemistry and Research Institute for Natural Sciences Korea University Seoul 02841 Republic of Korea; ^2^ Korea Basic Science Institute (KBSI) Seoul 02841 Republic of Korea

**Keywords:** core/shell, deep‐blue, high brightness, light‐emitting diodes, metal–halide perovskites

## Abstract

Metal–halide perovskite nanocrystals (NCs) have emerged as suitable light‐emitting materials for light‐emitting diodes (LEDs) and other practical applications. However, LEDs with perovskite NCs undergo environment‐induced and ion‐migration‐induced structural degradation during operation; therefore, novel NC design concepts, such as hermetic sealing of the perovskite NCs, are required. Thus far, viable synthetic conditions to form a robust and hermetic semiconducting shell on perovskite NCs have been rarely reported for LED applications because of the difficulties in the delicate engineering of encapsulation techniques. Herein, a highly bright and durable deep‐blue perovskite LED (PeLED) formed by hermetically sealing perovskite NCs with epitaxial ZnS shells is reported. This shell protects the perovskite NCs from the environment, facilitates charge injection/transport, and effectively suppresses interparticle ion migration during the LED operation, resulting in exceptional brightness (2916 cd m^−2^) at 451 nm and a high external quantum efficiency of 1.32%. Furthermore, even in the unencapsulated state, the LED shows a long operational lifetime (T_50_) of 1192 s (≈20 min) in the air. These results demonstrate that the epitaxial and hermetic encapsulation of perovskite NCs is a powerful strategy for fabricating high‐performance deep‐blue‐emitting PeLEDs.

## Graphical abstract

An effective perovskite encapsulation strategy is developed that enables the successful synthesis of deep‐blue‐emitting CsPb(Br_1‐x_Cl_x_)_3_/ZnS core/shell NCs. The epitaxial ZnS shell protects the perovskite NC from the environment, facilitates charge injection/transport, and effectively suppresses the interparticle ion migration during LED operation, achieving exceptional brightness (2916 cd m^‐2^) at 451 nm and high external quantum efficiency (EQE) of 1.32%.

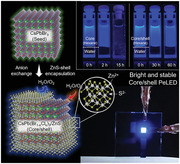

## 1 Introduction

Metal–halide perovskite nanocrystals (NCs), extensively studied for photovoltaic devices, have emerged as a highly promising class of materials for use in next‐generation light‐emitting diodes (LEDs). The excellent optoelectronic properties of perovskite NCs, including high photoluminescence quantum yields (PLQYs), facilely tunable bandgaps, narrow emission spectra, excellent charge‐transport properties, and low fabrication costs have spearheaded the development of perovskite‐based LEDs (PeLEDs).^[^ ^1^, ^2^, ^3^, ^4^ ^]^ Ligand selection, composition control, and heterostructure design have been commonly employed for the surface engineering of perovskite NCs to reduce surface defects and confine charge carriers. Therefore, state‐of‐the‐art red and green‐light‐emitting perovskite NCs now exhibit an outstanding external quantum efficiency (EQE) of over 20%^[^ ^5^, ^6^, ^7^, ^8^, ^9^, ^10^, ^11^, ^12^, ^13^, ^14^ ^]^ and blue and sky‐blue‐emitting perovskite NCs exhibit a commendable EQE of over 10%.^[^ ^14^, ^15^, ^16^, ^17^, ^18^, ^19^ ^]^

Deep‐blue LEDs are crucial to energy‐efficient solid‐state lighting and vivid color displays.^[^ ^20^ ^]^ Despite the considerable interest in deep‐blue PeLEDs, the inherently labile nature and large bandgap of deep‐blue perovskites^[^ ^21^, ^22^ ^]^ result in an unwanted electric‐field‐induced ion migration, which is detrimental to device performance, leading to low brightness and short lifetimes of deep‐blue PeLEDs.^[^ ^14^, ^15^, ^16^, ^17^, ^18^ ^]^ Therefore, the realization of high‐performance deep‐blue PeLEDs based on perovskite NCs requires a new surface engineering and design concept that forestalls the daunting problem of ion migration during operation.

Hermetically sealing the perovskite NCs is a conceptually straightforward strategy to fundamentally solve the dual problems of environmental sensitivity and interparticle ion migration during LED operations. However, forming a core/shell structure with epitaxial interfaces of core (perovskite) and shell components is a significant challenge if anion exchange is proceeded while the encapsulation reaction. Because of such anion exchanges, the core surface is rendered highly unstable, and the epitaxial shell growth step becomes complicated.^[^ ^23^, ^24^ ^]^ Notably, coexisting Br^–^ and Cl^–^ anions are required for developing deep‐blue perovskite NCs.^[^ ^21^, ^25^ ^]^ To achieve the deep‐blue emission color, controlling the balance of anions in the core perovskite NC of a core/shell system is essential. Furthermore, the deep‐blue‐emissive perovskite core inevitably requires a wide‐bandgap shell, such as ZnS, to confine the electron/hole within the core region effectively and enhance the radiative recombination (type I).^[^ ^26^, ^27^, ^28^, ^29^ ^]^ However, actually depositing the semiconductor shell epitaxially on the core perovskite NCs, while maintaining the latter in a defect‐free state with a complete hermetic sealing, is a formidable challenge. To deposit a ZnS shell, an effective route needs to be developed to stabilize the anion composition in the perovskite core and simultaneously prime the surface with Zn^2+^ cations for the further growth of ZnS.

In this study, we demonstrated that the anion exchange reaction of green‐emitting CsPbBr_3_ seed NCs with mixed‐halide ZnX_2_ (X = Cl, Br) additives leads to the formation of surface‐primed defect‐free deep‐blue‐emissive CsPb(Br_1‐x_Cl_x_)_3_ NCs. Furthermore, we introduced sulfur*–*octadecene (S–ODE) solution, an activated sulfur precursor, that reacts with oleylamine to evolve reduced sulfur species such as H_2_S.^[^ ^30^, ^31^, ^32^ ^]^ The subsequent reaction leads to the formation of a core/shell CsPb(Br_1‐x_Cl_x_)_3_/ZnS with a small lattice mismatch of only 8.5%. This structure shows a deep‐blue emission at 451 nm with a narrow bandwidth. The thin Zn‐based layer initially formed epitaxially on the anion‐passivated surface of the CsPb(Br_1‐x_Cl_x_)_3_ NCs allows further successful epitaxial growth of a robust and hermetic ZnS shell. The resulting core/shell CsPb(Br_1‐x_Cl_x_)_3_/ZnS NCs were employed to fabricate deep‐blue PeLEDs with a high brightness and long lifetime. The deep‐blue PeLEDs based on the core/shell CsPb(Br_1‐x_Cl_x_)_3_/ZnS NCs exhibited the maximum luminance (*L*_max_) with a remarkably high brightness of 2916 cd m^−2^, a considerable EQE of 1.32%, and high color purity (full width at half maximum, FWHM = 17.8 nm) with a deep‐blue emission at 451 nm. Further, the unencapsulated deep‐blue PeLEDs exhibited a record‐long operational half‐lifetime of 1185 s (≈20 min) in the air at an initial brightness of 100 cd m^−2^. Therefore, our work clearly demonstrates that an optimal epitaxial juxtaposition of a perovskite NC core with robust semiconductor shell is a viable strategy for realizing deep‐blue PeLEDs by successfully suppressing ion migration and retaining the structural integrity of perovskite NCs during operation.

## 2 Result and Discussion

### 2.1 Formation and Characterization of Deep‐Blue‐Emitting Core/Shell Perovskite NCs

**Figure 1:**
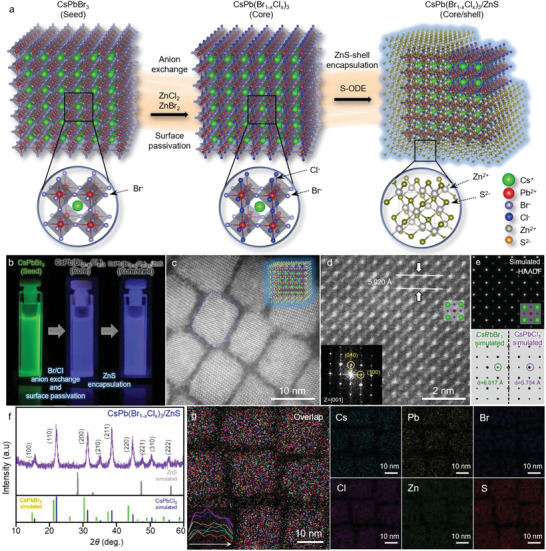
Characterization of the core/shell CsPb(Br_1‐x_Cl_x_)_3_/ZnS NCs. a) Schematic illustration of the synthesis of the core/shell NCs from the CsPbBr_3_ seed NCs. b) Photograph showing the change in PL color from that of the CsPbBr_3_ seeds (green) to that of the core CsPb(Br_1‐x_Cl_x_)_3_ (deep blue) and finally to that of the core/shell CsPb(Br_1‐x_Cl_x_)_3_/ZnS NCs (deep blue) through anion exchange and ZnS encapsulation; c) Representative atomic‐resolution HAADF–STEM image of core/shell NCs. d) Atomic‐resolution HRSTEM image with FFT pattern of core/shell NCs. e) Corresponding simulated atomic‐resolution HAADF–STEM image of core/shell NCs and FFT patterns of CsPbBr_3_ and CsPbCl_3_, respectively. f) PXRD patterns of the (i) core/shell NCs in comparison with the simulated patterns of the z–ZnS, CsPbBr_3_, and CsPbCl_3_ phases. g) The EDS elemental mapping analysis of the core/shell NCs.

**Figure** **1**a displays the synthetic scheme used to fabricate the deep‐blue‐emitting core/shell CsPb(Br_1‐x_C_x_)_3_/ZnS NCs (hereafter, core/shell NCs, Figure S1 (Supporting Information) and the Methods for details). The synthesis consists of three steps, viz. the formation of a deep‐blue CsPb(Br_1‐x_C_x_)_3_ core (hereafter, core NCs) via anion exchange, surface treatment via halide passivation, and epitaxial growth of the ZnS shell. Figure 1b displays the transformation of the green‐emitting CsPbBr_3_ at an emission wavelength of 515 nm to a deep‐blue emitting core/shell NCs (451 nm) (Figure S2, Supporting Information). Figure 1c and Figure S3a,b (Supporting Information) show the high‐angle annular dark‐field scanning transmission electron microscopy (HAADF–STEM) and transmission electron microscopy (TEM) images that confirm the formation of the core/shell structures. The core/shell NCs have an average size of 17.6 ± 1.73 nm with a narrow size distribution of less than 10%, which is larger than that of the CsPbBr_3_ seed NCs (14.6 ± 1.66 nm) (Figure S3c, Supporting Information). The high–resolution (HR)STEM image and fast Fourier transform (FFT) pattern reveal that Cl^–^ has been incorporated into the CsPbBr_3_ crystal structure (Figure 1d,e). When compared with the respective simulated results, the experimental lattice distance of the core (*d* = 5.920 Å) is shorter than that of the simulated lattice distance of CsPbBr_3_ (*d* = 6.017 Å). The smaller lattice distance of the core NCs, that arises after anion exchange, was confirmed by powder X‐ray diffraction (PXRD) analysis (Figure S4, Supporting Information), and the PXRD analysis was in agreement with those of the HRTEM and HRSTEM analyses. Markedly, the absence of the diffraction peaks of ZnS in the PXRD pattern of the core/shell NCs (Figure 1f) indicates the formation of a very thin ZnS shell that does not contain other crystalline impurities.^[^ ^33^, ^34^ ^]^ X‐ray photoelectron spectroscopy (XPS) confirmed the incorporation of mixed Cl^−^/Br^−^ and the formation of nano‐thin ZnS shells on the core/shell NCs (Figure S5, Supporting Information). In addition, energy‐dispersive X‐ray spectroscopy (EDS) mapping analyses (Figure 1g) revealed the uniform elemental distribution of the mixed‐halide core and ZnS shell.

The poor stability and intrinsic surface defects of the perovskite NCs pose a serious experimental difficulty that hinders the facile formation of the core/shell structures.^[^ ^35^, ^36^ ^]^ Figure S6 (Supporting Information) displays the destroyed structure of CsPbBr_3_ seed NCs, in which black dots have appeared due to the presence of reduced Pb^0^ (Figure S7a,b, Supporting Information); the structural degradation of the CsPbBr_3_ seed NCs, induced by exposure to electron beam irradiation, clearly shows the intrinsically poor stability of the pristine perovskite NCs and weak surface protection by halide ligands (Figure S7c,d, Supporting Information).

Three strict criteria of band alignment, surface passivation, and minimal lattice mismatch must be met for the shell material to successfully enclose the perovskites core without compromising the initial optoelectronic properties. When mixed ZnX_2_ (X = Cl and Br) halide additives were added, the control of halide composition and suppression of the surface defects of core NCs were simultaneously achieved to lead to successful color tuning and epitaxial ZnS growth (Figure S8, Supporting Information). For comparison, we prepared a CsPb(Br_1‐x_Cl_x_)_3_ NCs via the conventional direct (one‐pot) synthesis method^[^ ^25^ ^]^ and core NCs, which were treated with ZnX_2_ but not with S–ODE. As evidenced by the HRTEM analysis (Figure S9, Supporting Information), which shows a defect‐free crystalline surface of the core NCs, the smooth crystalline surface of core NCs allowed the facile in situ epitaxial growth of the ZnS shell (Figure S10, Supporting Information). The sulfur precursor should be judiciously chosen by considering its reactivity with the CsPbBr_3_ seed NCs, to ensure successful encapsulation of the perovskite and not to induce any damage to the crystal structure of the core.

**Figure 2:**
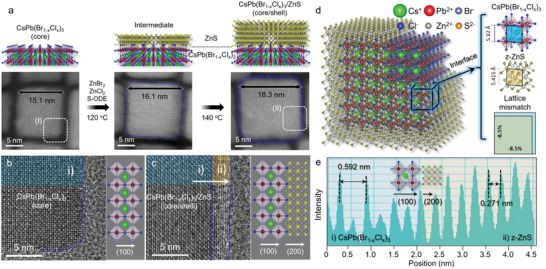
Epitaxial growth of the z–ZnS shell on the core CsPb(Br_1‐x_Cl_x_)_3_ NCs. a) Schematic illustration of the synthesis of the core NCs, intermediates, and core/shell NCs, with the corresponding HAADF–STEM images. Anion exchange with the ZnX_2_ additives and further ZnS shell growth with S–ODE through post‐treatment of the CsPbBr_3_ seed NCs increased the size of the NCs from 15.1 to 18.3 nm. b,c) Atomic‐resolution HRTEM images of the core and core/shell NCs, respectively, clearly showing the epitaxial growth of ZnS in region (ii) on the core region (i); Inset, atomic models according to the HRTEM analysis. d) 3D simulated atomic model of the core/shell NCs with lattice mismatch analysis of the epitaxial interface between core (100) and ZnS (200). e) Intensity profile determined from the yellow arrow in (c), indicating the respective lattice distances of (100) CsPb(Br_1‐x_Cl_x_)_3_ and (200) z–ZnS.

The outcome of the encapsulation process is heavily reliant on the intricate interplay of various reaction kinetics with the reactivity of sulfur precursors. The kinetically controlled reaction, with S–ODE and residual Zn^2+^ at 120–140 °C, enabled the formation of the ZnS shell on the surface‐passivated, damage‐free core NCs. However, when less reactive sulfur precursors, such as sulfur*–*trioctylphosphine (S–TOP) and 1‐dodecanethiol (1‐DDT) were used in the reaction,^[^ ^34^, ^37^, ^38^ ^]^ any significant structural changes or ZnS composition were not observed by PXRD (Figure S11, Supporting Information) and EDS analysis. Conversely, when a different sulfur precursor with a higher reactivity, viz. sulfur–oleylamine (S–OAm),^[^ ^37^ ^]^ was employed, some structural damage occurred on the core NCs, leading to the formation of PbS impurities (Figure S12, Supporting Information). The fast reaction between the active sulfur and Pb^2+^ is probably the underlying cause for these observations.^[^ ^27^ ^]^ These results highlight the importance of carefully selecting sulfur precursors in the encapsulation of core NCs and the potential trade‐offs between reactivity and structural stability. The quality of the ZnS shell could be further honed by adjusting the reaction temperature. Compared to the core/shell intermediate synthesized at the encapsulation temperature of 120 °C, a thicker ZnS shell with a smoother surface was obtained by the additional encapsulation step at 140 °C (**Figure** **2**a; Figure S13, Supporting Information). However, at a higher temperature of 150 °C, there was a definite disintegration of the crystal structure of the core NCs, which was accompanied by the accumulation of PbS impurities (Figure S14, Supporting Information).

The HRTEM images of the core and core/shell NCs (Figure 2b,c) show that both cores (highlighted in blue, region i) exhibit well‐resolved lattice fringes with a *d* spacing of 0.592 nm, which corresponds to the *d* spacing of the CsPb(Br_1‐x_Cl_x_)_3_ cubic phase along the (100) crystal facets. In contrast, a distinct heterointerface between core NCs and zinc blende ZnS (z–ZnS) shell was observed in Figure 2c (highlighted in yellow, region ii), in which the *d* spacing of 0.271 nm can be indexed to the (200) planes of z–ZnS. It has been reported that a lattice mismatch (8.5%) less than 15% between the core and shell should be maintained to allow for the epitaxial growth of a shell with an optimal thickness.^[^ ^27^, ^39^ ^]^ This requirement poses a challenge to the preparation of a core/shell structure with perovskites as the core. Figure 2d,e illustrates the 3D simulated atomic model of core/shell NCs with a lattice mismatch analysis and an intensity profile that was obtained from the highlighted region shown in Figure 2c. To confirm the epitaxial attachment of the two phases that comprised similar lattice parameters, we extended our investigation to obtain the most suitable atomic orientations of z–ZnS and cubic‐CsPb(Br_1‐x_C_x_)_3_ phase according to the HRTEM analysis. Two feasible z–ZnS orientations along the (100) zone axis were identified, based on the cubic sub‐unit structure of CsPb(Br_1‐x_C_x_)_3_ phase, leading to the combined projected crystal structure models along the (200) and (100) directions of the z–ZnS and CsPb(Br_1‐x_Cl_x_)_3_, respectively (Figure S15a, Supporting Information), with the calculated lattice mismatches (Figure S15b–d, Supporting Information). Notably, the overlapped atomic models of the CsPb(Br_1‐x_C_x_)_3_ (100) and unrotated z–ZnS (200) directions exhibit a minimal lattice mismatch (8.5%), which is suitable for the formation of a ZnS shell structure on the CsPb(Br_1‐x_C_x_)_3_ core. The optimal band alignment structure of the wide bandgap of ZnS, the defect‐free surface of the deep‐blue emissive core NCs, and the minimal lattice mismatch of the synthesized core/shell NCs demonstrate that our method can be used as a rational synthetic scheme for producing defect‐free core NCs enclosed by the epitaxially grown ZnS shells.

**Figure 3:**
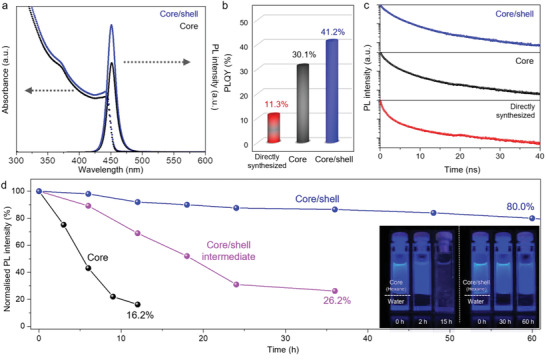
PL properties with thin film PLQY and chemical stability of the perovskite NCs. a) UV–vis absorption and PL spectra of the core and core/shell NCs. b) Thin film PLQY values of the directly synthesized CsPb(Br_1‐x_Cl_x_)_3_ NCs, core, and core/shell NCs. c) TRPL spectra. (*τ*_ave_  =  2.3, 1.2, and 0.60 ns for the core/shell, core, and directly synthesized CsPb(Br_1‐x_Cl_x_)_3_ NCs, respectively). d) Chemical stability test of the core, core/shell intermediate, and core/shell NCs, (inset) photographs of the core and core/shell NC solution exposed to water, taken under UV irradiation (*λ =* 365 nm) at different times; from left to right: core and core/shell NCs.

To explore the effect of the core/shell structure on the emission properties, we obtained the thin film PLQY and photoluminescence (PL) data. As shown in **Figure** **3**a,b, the surface defects of the directly synthesized CsPb(Br_1‐x_Cl_x_)_3_ NCs are associated with deep trap states and fragility; hence, the directly synthesized CsPb(Br_1‐x_Cl_x_)_3_ NCs exhibited a low PLQY (11.3%). The core NCs displayed a higher PLQY (30.1%), which may be attributed to the passivated surface of the core from the ZnX_2_ treatment. Notably, the measured PLQYs of the core/shell NCs are remarkably high (41.2%), which validate the positive roles played by the post‐treatment methods (ZnX_2_ treatment and encapsulation process) in the surface stabilization and passivation of the trap sites on the core.^[^ ^29^, ^40^ ^]^ Moreover, to gain further insight into the exciton recombination dynamics, we measured the time‐resolved PL spectra of a series of perovskite NCs at an excitation wavelength of 374 nm (Figure 3c). The three curves display similar spectral features and are well‐fitted by a multiexponential decay. In parallel with the PLQY results, core/shell NCs displayed a lower rate of emission decay than those displayed by the directly synthesized CsPb(Br_1‐x_Cl_x_)_3_ and core NCs. The core/shell NCs had the most extended PL lifetime among the three samples, indicating that the non‐radiative recombination is effectively suppressed (Table S1, Supporting Information).^[^ ^41^ ^]^

To investigate the role of the ZnS shell on the stability of the perovskites to environment, we performed a water soaking test by exposing the nanocrystals to a hostile environment. The core NCs lost 80% of its PL intensity after 12 h, which indicates the rapid desorption of the ligands and the severe structural disintegration of the perovskite NCs by the action of water molecules (Figure 3d; Figure S16, Supporting Information).^[^ ^22^, ^28^ ^]^ In contrast, the structural stability of core/shell NCs toward water was much higher than those of the core NCs and core/shell intermediates. The core/shell NCs maintained more than 80% of their initial PL intensity even after 60 h of the test, sustaining a bright deep‐blue PL emission (Figure 3e). The high chemical stability of the core/shell NCs validated the effective isolation of the labile perovskite core by the epitaxially grown ZnS, which is more robust and affords greater hermetic sealing than the alkyl ligands.

### 2.2 Deep‐Blue‐Emitting PeLED Architecture and Performance

**Figure 4:**
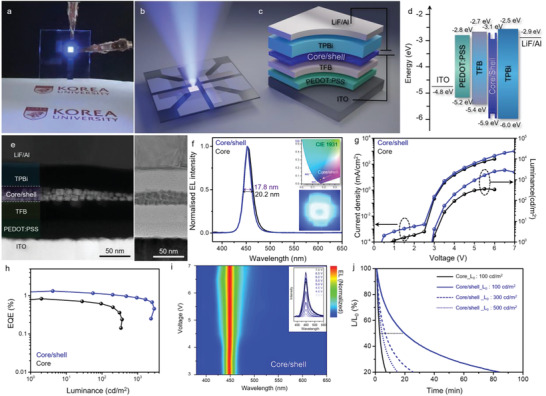
Fabrication of the PeLED and its performance evaluation. a) Photograph of the operating PeLED. b,c) Illustration of the deep‐blue emitting PeLED and the corresponding configuration of the PeLED cell, with PEDOT:PSS and TFB as the HTL and ETL, respectively. d) Energy‐band diagram of the PeLEDs. e) Cross‐sectional HAADF–STEM and HRTEM images of the focused‐ion‐beam‐milled core/shell PeLED. f) Electroluminescence spectra of the core and core/shell PeLEDs. Inset, CIE (Commission Internationale de l'Éclairage) color coordinates of the core/shell NCs and photograph of the operating device. g) Voltage‐dependent current density (left axis) and luminance (right axis) (*J–V–L*) curves of the core and core/shell PeLEDs. h) EQE–luminance (EQE–L) curves. i) Driven‐voltage‐dependent electroluminescence spectra of the deep‐blue PeLEDs based on core NCs. j) Operational lifetimes of the core and core/shell PeLEDs at different luminance values.

After understanding the photophysical properties of a series of perovskite NCs, we fabricated PeLEDs based on the configuration of glass/indium tin oxide (ITO)/poly(3,4–ethylenedioxythiophene) polystyrene sulfonate (PEDOT:PSS)/poly[(9,9–dioctylfluorenyl–2,7–diyl)–*co*–(4,4–(*N*–(4–sec–butylphenyl))) diphenylamine)(TFB)/perovskite NCs/TPBi/LiF/Al multilayer structure (**Figure** **4**a–d; Figure S17, Supporting Information). A HAADF–STEM and HRTEM images of the cross‐section of the fabricated PeLED based on the core/shell NCs (core/shell PeLED) shows a multilayer structure with well‐defined boundaries between each layer (Figure 4e). This cross‐sectional image clearly shows the thickness of each optimized layer including the uniform active emission layer of well‐dispersed core/shell NCs. Core/shell PeLEDs exhibit a very narrow FWHM of 17.8 nm and a symmetric electroluminescence emission peak at 451 nm that matches well with that of the PL emission band (Figure 4f; Figure S18, Supporting Information). The core/shell PeLED fabricated herein achieved an ideal deep‐blue emission for display applications by exhibiting CIE color coordinates of (0.17, 0.06).

The luminance and current density curves for the PeLEDs fabricated in this study are illustrated in Figure 4g, as functions of the applied voltage (*J*–*V*–*L* curves). Since ZnS, the shell material, has an energy level properly aligned with those of TFB as the hole transfer layer (HTL), deep‐blue‐emitting core/shell PeLEDs can realize lower turn‐on voltages (*V*_on_ = 2.88 V) than those realized by core NC‐based PeLED (core PeLEDs). Remarkably, the core/shell PeLEDs exhibited a maximum luminance of 2916 cd m^−2^ at a current density of 752.9 mA cm^−2^, which was more than eight times higher than that of the core PeLED (*L* = 366.2 cd m^−2^). This result can be attributed to the relatively high thin film PLQY (>40%) of the core/shell NCs and indicates a significantly more efficient charge injection/transport. In addition, the core/shell PeLEDs showed a maximum EQE of 1.32%, corresponding to a current efficiency of 2.74 cd A^−1^ and a power efficiency of 2.86 lm W^−1^, respectively. (Figure 4h; Figure S19 and Table S2, Supporting Information). The above device performance is unusually high for PeLEDs generating 451 nm emission and has not been achieved with other deep‐blue PeLEDs. (Figure S20 and Table S3, Supporting Information). The superior performance of this well‐defined core/shell PeLED is attributed to the relatively high PLQY achieved due to the elimination of defects in the core NCs during ZnX_2_ treatment. Furthermore, the n‐type semiconductor ZnS shell improves charge transport in the core NC, ensuring good charge balance in the light emissive layer (EML) (Figure S21, Supporting Information), because high‐quality core/shell NCs with very low defect density would suppress non‐radiative decay and reduce energy loss.^[^ ^29^ ^]^

The operational stability of deep‐blue PeLEDs in ambient air, which not only relies on the stability of the EMLs but is also highly dependent on the interfacial contacts and the charge injection balance in the devices, is crucial for practical applications.^[^ ^42^, ^43^, ^44^, ^45^ ^]^ Therefore, we performed operational lifetime tests of unencapsulated PeLEDs by applying a constant current and monitoring the corresponding luminance in the air at room temperature. Compared with the T_50_ of core PeLEDs (181 s) at the initial luminance (*L*_0_) = 100 cd m^−2^, core/shell PeLED exhibited a much longer T_50_ of over 1000 s (*T*_50_ = 1192 s) (Figure 4j). The operational lifetimes of core/shell PeLEDs were 407 and 278 s at *L*_0_ of 300 and 500 cd m^−2^_,_ respectively. The greatly improved operational stability of core/shell PeLEDs can be attributed to well‐defined thin EML, suppressed defect density, and mitigation of the ion migration by the hermetic sealing of the core by the ZnS shell. The operational stability of unencapsulated deep‐blue PeLEDs in ambient air is among the best of reported examples, when compared with blue PeLEDs with a CIE_y_ < 0.1 (Figure S22, Supporting Information). Notably, as the bias voltage is increased to 7 V, the electroluminescence peak position of core/shell PeLED remains constant with only marginal broadening, ascribed to the charge carrier/phonon interaction owing to Joule heating (Figure 4i; Table S4, Supporting Information).^[^ ^46^ ^]^ In contrast, the peak exhibited by the core PeLEDs drastically broadens and undergoes red shift; thus, the latter LED loses its original deep‐blue emitting ability (Figure S23, Supporting Information). Furthermore, the change in the EL spectrum also showed a similar trend as the operation time increased (Figure S24, Supporting Information). Considering the improved charge balance inside the core/shell NC, the broadened exciton recombination zone in the EML is conducive to the excellent efficiency and operational stability of PeLEDs.^[^ ^47^, ^48^ ^]^ This observation confirms the key role played by the ZnS shell in the performance of core/shell PeLEDs.

**Figure 5:**
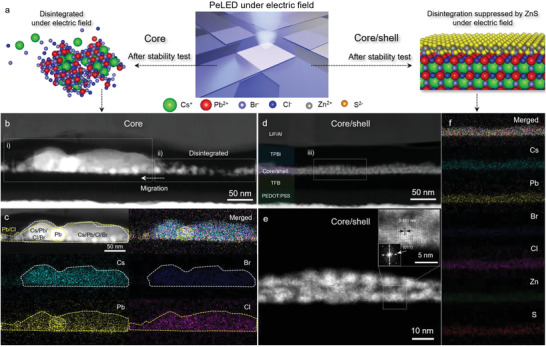
Operational stability of the core and core/shell PeLED devices. a) Schematic illustration of the ion‐migration‐suppressed core/shell under an electric field, compared with that of core CsPb(Br_1‐x_C_x_)_3_. b,d) Cross‐sectional HAADF–STEM image of the focused‐ion‐beam‐milled core and core/shell PeLED devices after the stability test (*L*/*L*_0_ *=* 50%). c) EDS elemental maps of Cs, Pb, Br, and Cl for the core NCs, obtained from the area enclosed by the white dashed box (i). e) Highly magnified HAADF–STEM image and HRSTEM analysis result (inset) of the core/shell NCs in the PeLED after the stability test. f) EDS elemental maps of Cs, Pb, Br, Cl, Zn, and S for the core/shell NCs, obtained from the area enclosed by the white dashed box (iii).

To further elucidate the mechanism of the operational stability of the PeLEDs, we obtained HAADF–STEM and corresponding EDS mapping images of each EML from the representative core and core/shell PeLED obtained after the stability test (*L*/*L*_0_ = 50%), respectively (**Figure** **5**a). The EML in the core PeLED did not retain its initial morphology due to the severe disintegration of the core NCs (Figure 5b,c). Nanocrystal merging and ion migration of the metal (Cs, Pb) and halide (Cl, Br) ions occurred to cause bandgap shift and thus a substantial efficiency roll‐off.^[^ ^49^, ^50^ ^]^ Conversely, the initial morphology of the core/shell NCs‐based EML shows a negligible change, and the crystallinity of the initial core/shell NCs remains intact after the operation of the device (Figure 5d–f). Even after harsher stability tests (*L*/*L*_0_ < 20%), EML morphology of core/shell PeLED is mostly retained due to significantly reduced ion migration as compared to that of core NCs (Figure S25, Supporting Information). Therefore, it is evident that the ion migration and phase separation of the perovskite NCs are the main reasons for the deterioration of the performance of the conventional PeLEDs. Our study unambiguously demonstrates that the emergence of a perovskite‐based core/shell system, comprising a robust inorganic shell in the form of ZnS, points to a viable solution toward practical deep‐blue PeLED devices.

## 3 Conclusion

We developed an effective perovskite encapsulation strategy that enables the controllable synthesis of deep‐blue‐emitting core/shell NCs. We demonstrated that color tuning and elimination of surface defects of the core NCs can be simultaneously achieved by the addition of ZnX_2_. Surface priming with ZnX_2_ was critical for maintaining the anion balance of the core as well as for providing a primer layer for further ZnS growth. The epitaxial growth of ZnS was accomplished with a subsequent hot‐injection of S–ODE and further aging. The small lattice mismatch between the core CsPb(Br_1‐x_Cl_x_)_3_ crystal and the shell ZnS NCs was critical to the successful epitaxial growth of the ZnS phase. The hermetic sealing of the core component by the ZnS shell protected the core NCs from the environment and occluded ion migration under an electric field to improve the optical/photophysical performance and stability of the PeLEDs. The observed excellent brightness (2916 cd m^−2^), EQE value (1.32%), and notable operational half‐lifetime of ≈20 min at an initial brightness of 100 cd m^−2^ were among the best reported parameters of deep‐blue emitting PeLEDs. We expect that the CsPb(Br_1‐x_Cl_x_)_3_/ZnS NCs analogues synthesized with the herein reported design concept of hermetic sealing and surface engineering, assisted by surface priming of the cation in the semiconductor for the epitaxial growth of the shell, will spearhead further advancements in next‐generation PeLED display and lighting technologies.

## 4 Experimental Section

### Chemicals

Cesium carbonate (Cs_2_CO_3_, 99.9%), lead (II) bromide (PbBr_2_), oleic acid (OA, technical grade 90%), oleylamine (OAm, technical grade 70%), 1‐octadecene (ODE, technical grade 90%), 1‐dodecanethiol (1‐DDT, ≥ 98%), trioctylphosphine (TOP, 97%), zinc bromide (ZnBr_2,_ 99.999%), zinc chloride (ZnCl_2_, reagent grade, ≥ 98%), sulfur powder, and all solvents, including toluene (anhydrous 99.8%), ethyl acetate (EtOAc, anhydrous 99.5%), and hexane (reagent grade ≥ 95%) were purchased from Sigma–Aldrich. PEDOT:PSS; Clevios P VP Al4083 was purchased from Semiphion Company. Poly[(9,9‐dioctylfluorenyl‐2,7‐diyl)‐co‐(4,4′(N‐(4‐sec‐butylphenyl))) diphenylamine)] (TFB) was purchased from American Dye Source, Inc. Al was purchased from Aldrich. 2,2′,2″‐(1,3,5‐Benzinetriyl)‐tris(1‐phenyl‐1H‐benzimidazole) (TPBi) was purchased from ENanoTec Co., Ltd.

### Preparation of Precursor Solutions

All the chemicals were treated under an inert atmosphere. For the preparation of the cesium oleate solution, Cs_2_CO_3_ (0.814 g), ODE (40 mL), and OA (4 mL) were prepared in a three‐neck round bottom flask (100 mL), equipped with a magnetic stirring bar^3^. The flask and its contents were transferred to a preheated (120 °C) oil bath under vacuum for 1 h and thereafter maintained under Ar at 150 °C until all the Cs_2_CO_3_ had reacted with OA. The solution was cooled to room temperature (25 °C) for storage, and preheated to 100 °C before use. For the preparation of the S–ODE solution, sulfur powder (1.28 g) and ODE (100 mL) were transferred into a two‐neck round bottom flask (250 mL) and maintained under vacuum for 30 min at room temperature to eliminate the oxygen. Thereafter, the mixture was heated to 190 °C for 30 min under Ar atmosphere. The solution was then cooled to room temperature. All the precursors were stored under Ar atmosphere before use.

### Synthesis of Green‐Emitting CsPbBr_3_ Seed NCs

CsPbBr_3_ NCs were synthesized using a previously reported hot‐injection method.^[^ ^25^ ^]^ PbBr_2_ (0.140 g), ODE (10 mL), OA (1 mL), and OAm (1 mL) were transferred to a three‐neck round bottom flask (100 mL) and dried for 1 h at 120°C under vacuum. The mixture was subsequently heated to 180 °C under Ar, until a clear solution was obtained. Thereafter, the preheated Cs–oleate solution (1 mL) was injected into the mixture, and 10 s later, the mixture was cooled by immersion into an ice‐water bath. For the purification of the CsPbBr_3_ NCs, the crude solution was first precipitated by centrifugation at 7000 rpm for 5 min. After centrifugation, the precipitates were dispersed in ODE (10 mL) for further use.

### Synthesis of Deep‐Blue Emitting Core/Shell CsPb(Br_1‐x_Cl_x_)_3_/ZnS NCs (Core/Shell NCs)

For the synthesis of the core/shell NCs (Figure S1, Supporting Information), ZnCl_2_ (0.082 g), ZnBr_2_ (0.045 g), ODE (10 mL), and OAm (0.5 mL) were transferred into a three‐neck round bottom flask (100 mL) and dried under vacuum at 120 °C for 10 min. The mixture was placed under Ar for 10 min. CsPbBr_3_ seed solution (10 mL) and S–ODE (1.0 mL) (core NCs were synthesized without the addition of S–ODE) were quickly added and kept for 30 min. Thereafter, the additional reaction step was performed at 140 °C. After the reaction had proceeded for 20 min, the solution was cooled to room temperature using an ice‐water bath. For the purification of the NCs, the crude solution was first precipitated by centrifugation at 7000 rpm for 5 min. After centrifugation, the precipitates were dispersed in toluene (10 mL) and washed again with an equal volume of EA. The precipitates were centrifuged at 10 000 rpm for 10 min for further use or device fabrication.

### Characterization

TEM and HRTEM images were captured using TECNAI G2 F30 ST (300 kV) and TECNAI G2 20 S–Twin (200 kV) devices, respectively. EDS data were recorded using the energy‐dispersive X‐ray spectrometer contained in the TECNAI G2 F30 ST. High‐spatial‐resolution EDS analysis and HAADF–STEM were performed at the FEI Nanoport in Eindhoven using a Titan Probe Cs TEM 300 kV with Chemi–STEM technology. EDS elemental mapping results for the intermediates were obtained using a Super–X detector with an X–FEG module. XRD patterns were obtained using a Rigaku Ultima III diffractometer system with a graphite‐monochromatized Cu K*α* radiation source operating at 40 kV and 40 mA. The Visualization for Electronic and Structural Analysis (VESTA 64bit) and SingleCrystal programs were used to simulate the XRD and FFT patterns, respectively. XPS was performed using a K–ALPHA+ XPS system (Thermo Fisher Scientific, UK) equipped with monochromatic Al K*α* (1486.6 eV) radiation. The ultraviolet photoelectron spectroscopy (UPS) was performed using a Nexsa XPS system (Thermo Fisher Scientific, UK) equipped with He‐I (21.22 eV) as the UV source. The absorption and PL spectra of the perovskite NC solutions were measured using an UV–visible spectrometer (HP 8453, *λ* = 190–1100 nm) and fluorescence spectrophotometer (Hitachi F–7000) equipped with an integrated sphere (ILF–835). A JASCO FP–8500 fluorescence spectrometer, equipped with an integrated sphere was utilized to measure the PLQY for the perovskite film. The PL spectrum of the perovskite NCs solution was collected at room temperature over the wavelength range of 300–800 nm under a xenon lamp excitation of 150 W (wavelength of 450 nm; wavelength range: from −20 to +20 nm). The slit width was fixed at 0.6 nm and the data was accumulated four times to generate an average value. Time‐resolved fluorescence (TRF) signals of all the samples were measured using the time‐correlated single‐photon counting method. The samples were excited using a 375‐nm pulse and the TRF signals were measured at 448 nm using a photomultiplier tube (PMT, PMA182, PicoQuant). The TRF signals were fitted by a multiexponential function, and the average lifetime was determined by Equation 1:

(1) $$\tau_{\mathrm{avg}}=\frac{\sum_{i}A_{i}\tau_{i}}{\sum_{i}A_{i}}$$

where A_i_ and *τ*_i_ represent the amplitude and decay time, respectively, of the individual components for the multiexponential decay profiles.

### PeLED Device Fabrication and Characterization

The PeLED device was fabricated on a glass substrate (sheet resistance = 10 Ω m^−2^) and subsequently coated with an anode and a transparent ITO layer; the dimensions of the active pattern were 2 mm × 2 mm. The glass substrate was washed with distilled water in an ultrasonicator for 10 min, washed with high‐performance liquid chromatography (HPLC) grade isopropanol for 20 min, and dried overnight in an oven at 120 °C. PEDOT:PSS was spin‐coated on the ITO after UV/ozone (UVO) treatment for 20 min on the post‐treated glass substrate, and heat‐treated at 155 °C for 15 min to form a hole injection layer (30 nm). Thereafter, TFB, a hole transporting material, dissolved in chlorobenzene, was spin‐coated and annealed at 122 °C for 20 min to form an HTL (30 nm). perovskite NCs dissolved in octane solvent (≈10 mg mL^−1^) were spin‐coated to form a perovskite layer (20 nm). The electron transport layer (ETL), i.e., TPBi (40 nm), the electron injection layer, i.e., LiF (1 nm), and the cathode, i.e., Al (100 nm), were thermally deposited under vacuum (pressure < 5 × 10^−6^ Torr). The current density–voltage (*J–V*) and luminance–voltage (*L–V*) of the fabricated PeLED device were measured using Keithley 237 SMU and Konica Minolta CS–100A spectroradiometer, respectively. Electroluminescence spectra and CIE 1931 color coordinates were obtained using a Konica Minolta CS–2000 spectroradiometer. All the electroluminescence and device characteristics of the unencapsulated devices were measured in air at room temperature.

## Conflict of Interest

The authors declare no conflict of interest.

## Supporting information

Supporting Information

## Data Availability

The data that support the findings of this study are available from the corresponding author upon reasonable request.
