# Supplementary material for: Perovskite Nanocrystals Protected by Hermetically Sealing for Highly Bright and Stable Deep‐Blue Light‐Emitting Diodes
Source: Adv Sci (Weinh). 2023 Jun 4;10(23):2302906. doi: 10.1002/advs.202302906 (PMC10427390; doi:10.1002/advs.202302906)
Supplement: Supplementary file 1 — Supporting Information [file ADVS-10-2302906-s001.pdf]

ADVANCED SCIENCE
Open Access

Supporting Information

for *Adv. Sci.*, DOI 10.1002/advs.202302906

Perovskite Nanocrystals Protected by Hermetically Sealing for Highly Bright and Stable Deep-Blue Light-Emitting Diodes

Yongju Hong, Chungman Yu, Hyeondoo Je, Jin Young Park, Taekyung Kim, Hionsuck Baik, Gracita M. Tomboc, Youngseo Kim, Jung Min Ha, Jinwhan Joo, Chai Won Kim, Han Young Woo, Sungnam Park, Dong Hoon Choi and Kwangyeol Lee**

Supporting Information

Perovskite Nanocrystals Protected by Hermetically Sealing for Highly Bright and Stable Deep-Blue Light-Emitting Diodes

Yongju Hong, Chungman Yu, Hyeondoo Je, Jin Young Park, Taekyung Kim, Hionsuck Baik, Gracita M. Tomboc, Youngseo Kim, Jung Min Ha, Jinwhan Joo, Chai Won Kim, Han Young Woo, Sungnam Park, Dong Hoon Choi, and Kwangyeol Lee**

Dr. Y. Hong, C. Yu, H. Je, J. Y. Park, Dr. G. M. Tomboc, Dr. Y. Kim, J. M. Ha, Dr. J. Joo, C. W. Kim, Prof. H. Y. Woo, Prof. S. Park, Prof. D. H. Choi, Prof. K. Lee
Department of Chemistry and Research Institute for Natural Sciences, Korea University, Seoul 02841, Republic of Korea
E-mail: dhchoi8803@korea.ac.kr
E-mail: kylee1@korea.ac.kr

Dr. T. Kim, Dr. H. Baik,
Korea Basic Science Institute (KBSI), Seoul 02841, Republic of Korea

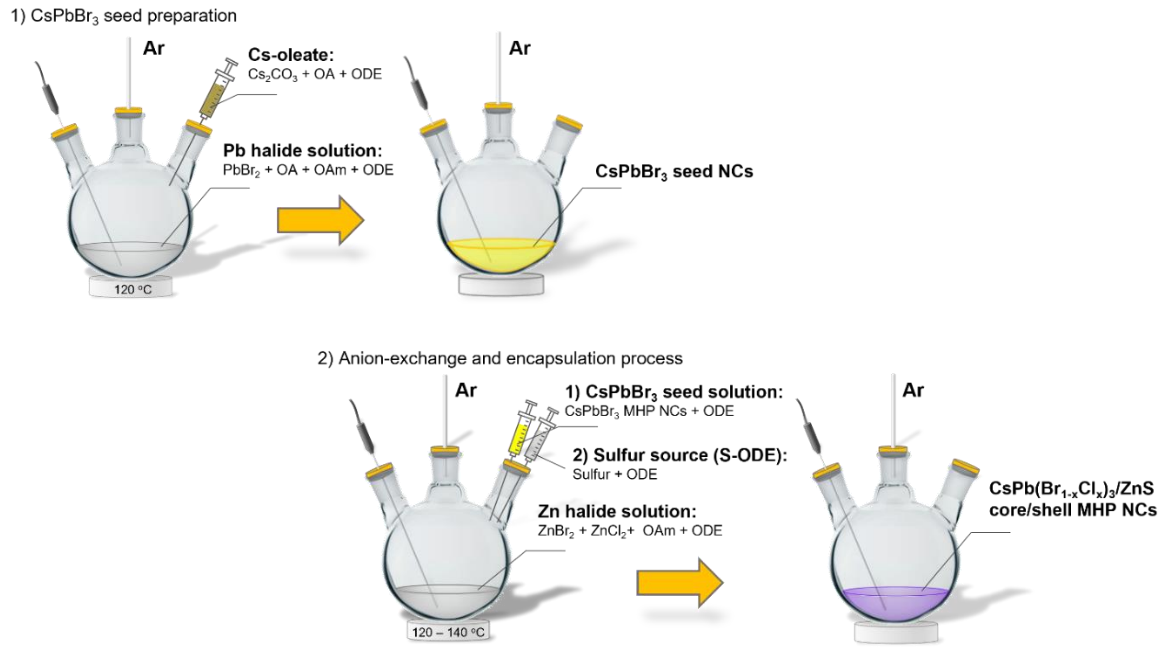

Figure S1. Schematic of the synthesis protocol. Steps included are as follows:
1) Synthesis of pristine $CsPbBr_3$ seed halide perovskite nanocrystals (NCs). 2) Anion-exchange and encapsulation for core/shell $CsPb(Br_{1-x}Cl_x)_3/ZnS$ NCs using a modified hot-injection and seed-mediated method.

Note S1. Steps involved in synthesis.

The synthesis consists of three steps:

(i) formation of the $CsPb(Br_{1-x}Cl_x)_3$ core *via* anion-exchange,

(ii) surface treatment *via* halide passivation, and

(iii) epitaxial growth of the ZnS shell.

The mixed-halide ZnX_2 (X = Cl, Br) additives functioned as both anion-exchange/surface passivation agents and Zn ion sources for the ZnS shell. Initially, the $CsPbBr_3$ seed NCs solution (in ODE) was injected into the pre-heated $ZnCl_2/ZnBr_2$ halide solution (in ODE) to minimize the heat exposure of $CsPbBr_3$ seeds and to avoid potential structural damage to the seeds. Thereafter, a rapid anion-exchange between Br^- and Cl^- occurred, forming a mixed-halide $CsPb(Br_{1-x}Cl_x)_3$ core. Simultaneously, the mixed-halide ZnX_2 served as a passivation agent for the as-treated $CsPb(Br_{1-x}Cl_x)_3$ surface that:

(1) eliminated the surface defects, which often lead to the formation of "black dots" on the perovskite surface, indicating a severe structural degradation, and

(2) served as a stable platform for the epitaxial growth of a thin ZnS shell, which is anticipated to hermetically seal the deep-blue-emitting $CsPb(Br_{1-x}Cl_x)_3$ cores to ensure

protection from the environment and to disable interparticle ion migration under an electric field. Finally, the reaction of Zn ions with S–ODE allowed the epitaxial growth of a thin ZnS shell to enclose the mixed-halide $CsPb(Br_{1-x}Cl_x)_3$ core.

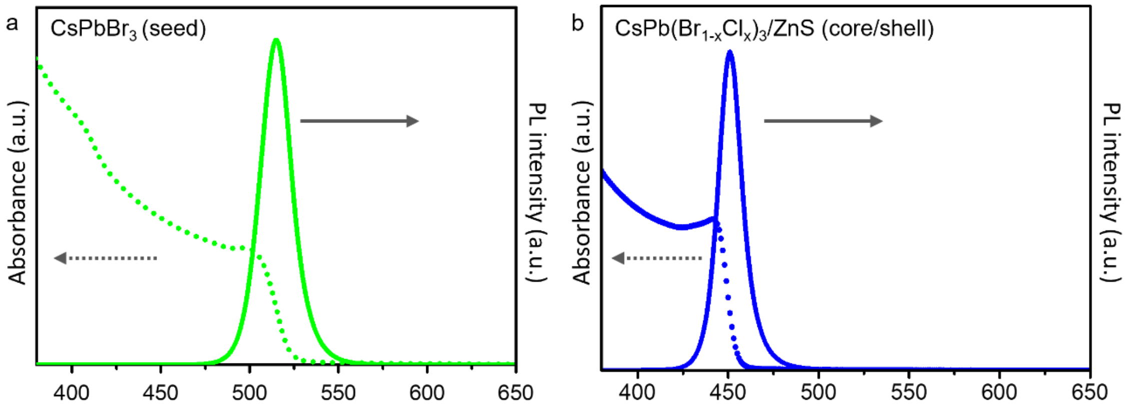

Figure S2. PL and UV–Vis absorption spectra. a, Green-emitting $CsPbBr_3$ seed NCs (λ_{em}^{max} = 515 nm) and **b,** deep-blue-emitting core/shell $CsPb(Br_{1-x}Cl_x)_3$/ZnS NCs (λ_{em}^{max} = 451 nm).

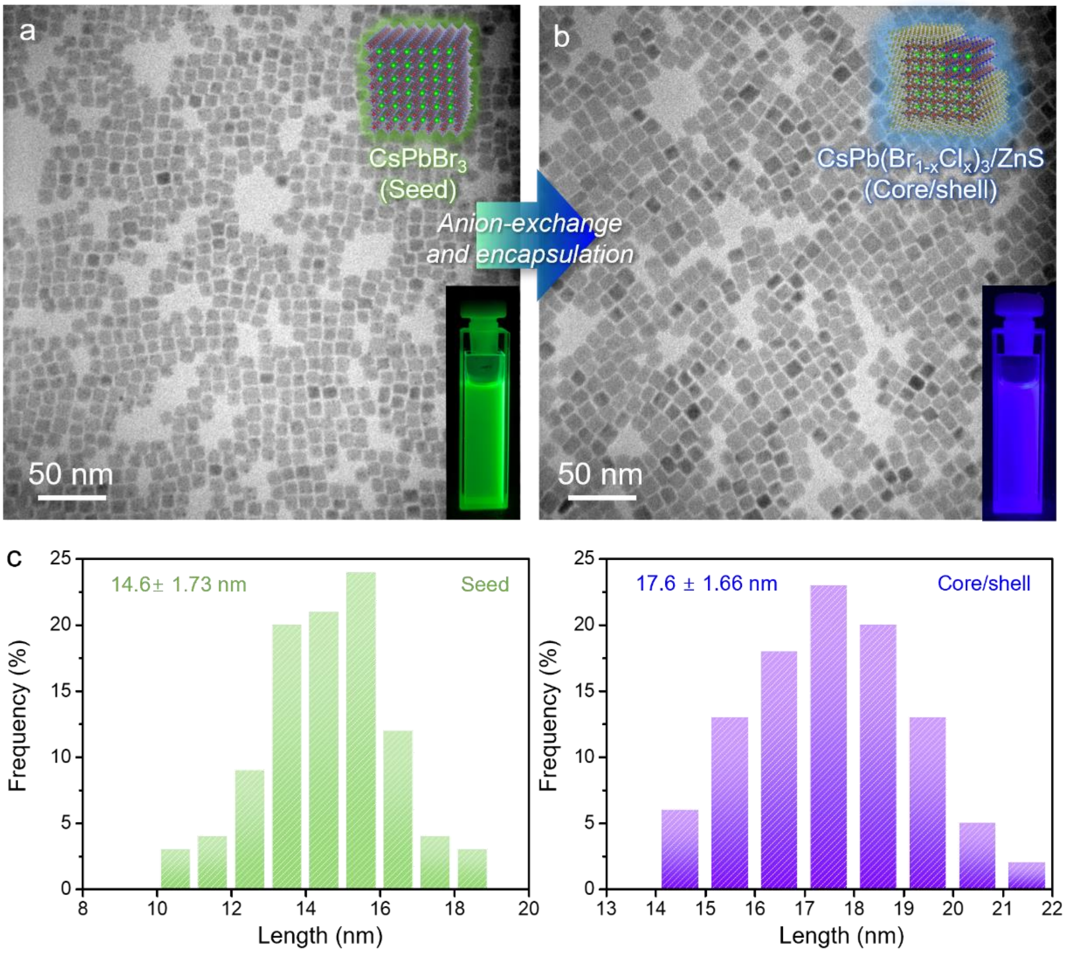

Figure S3. Representative TEM images and the corresponding histogram of size distribution. **a,** $CsPbBr_3$ seed NCs and **b,** Core/shell $CsPb(Br_{1-x}Cl_x)_3/ZnS$ NCs, respectively; **c,** size distribution diagrams of $CsPbBr_3$ and core/shell $CsPb(Br_{1-x}Cl_x)_3/ZnS$ NCs, respectively.

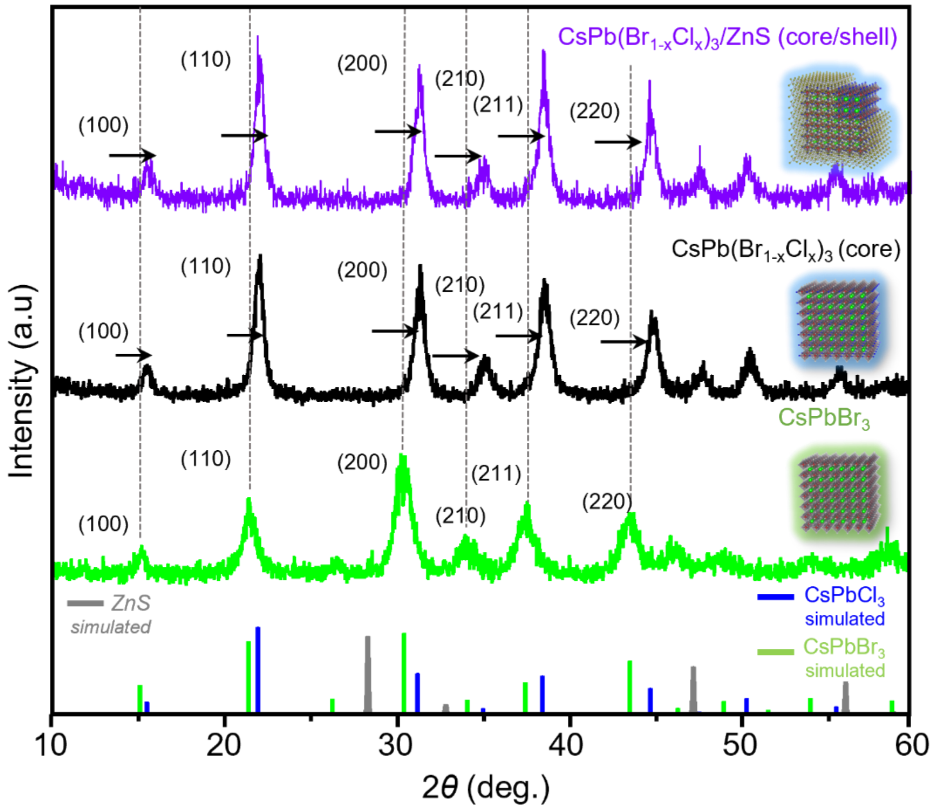

Figure S4. PXRD spectra of the pristine $CsPbBr_3$, $CsPb(Br_{1-x}Cl_x)_3$ core, and core/shell $CsPb(Br_{1-x}Cl_x)_3$/ZnS NCs. The PXRD spectra of the $CsPb(Br_{1-x}Cl_x)_3$ core and core/shell $CsPb(Br_{1-x}Cl_x)_3$/ZnS NCs indicate a slight right-shift of the peaks upon the facilitation of anion-exchange (incorporation of Cl^-), compared to the spectra of the $CsPbBr_3$ seed NCs.

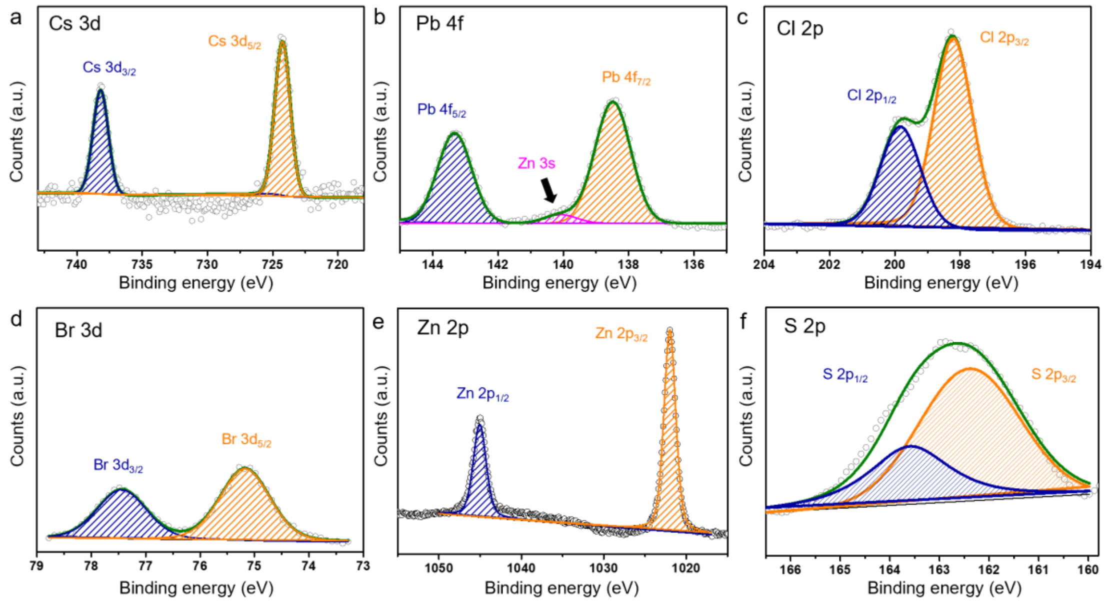

Figure S5. XPS profiles. a, Cs 3d; **b,** Pb 4f; **c,** Cl 2p; **d,** Br 3d; **e,** Zn 2p; and **f,** S 2p regions of core/shell $CsPb(Br_{1-x}Cl_x)_3/ZnS$ NCs.

Note S2. Comparison of the XPS spectra.

The XPS spectra of Cs 3d, Pb 4f, Cl 2p, Br 3d indicate the formation of the core $CsPb(Br_{1-x}C_x)_3$ NCs. Moreover, the Zn 2p and S 2p spectra of core/shell $CsPb(Br_{1-x}Cl_x)_3/ZnS$ NCs were similar to those of Zn^{2+} and S^{2-} of ZnS, respectively. The similarity further indicates the formation of nano-thin ZnS shells on the core/shell $CsPb(Br_{1-x}Cl_x)_3/ZnS$ NCs. Furthermore, in the Pb 4f region, the additional peak located at 139.87 eV corresponds to the Zn 3s that results from the ZnS shell.

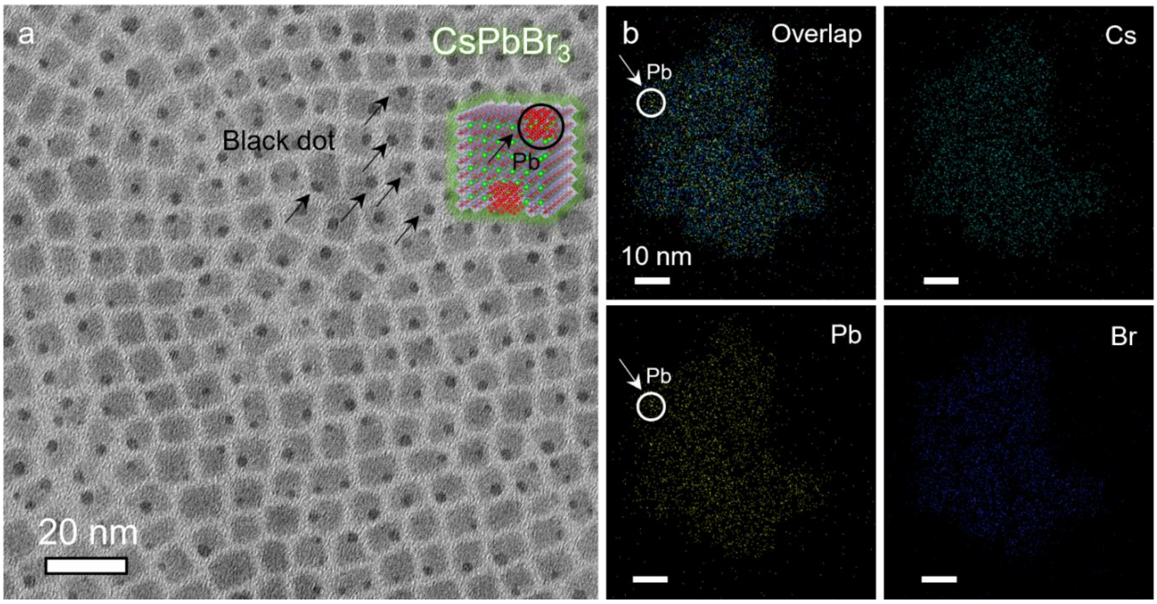

Figure S6. TEM characterization and EDS elemental analysis of $CsPbBr_3$ with black dots. **a,** TEM image of the pristine $CsPbBr_3$ seed NCs with black dots; **b,** EDS elemental mapping images demonstrating the chemical compositions of the $CsPbBr_3$ seed NCs and black dots.

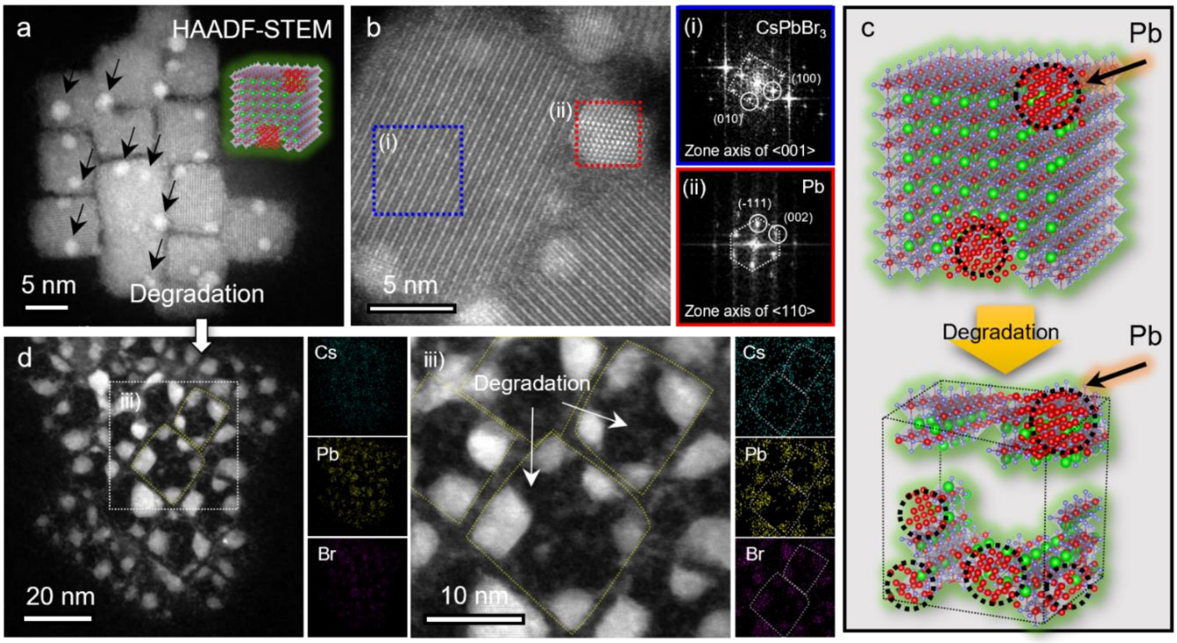

Figure S7. Morphology and crystal structure of the $CsPbBr_3$ seed NCs and degradation process. **a,** Representative HAADF–STEM image of the $CsPbBr_3$ seed NCs. b, Atomic-resolution HRSTEM image and the corresponding FFT patterns of regions (i) and (ii), which indicate $CsPbBr_3$ and metallic Pb, respectively. c, Schematic showing the degradation process of the $CsPbBr_3$ seed NCs induced by electron beam irradiation. d, HAADF–STEM image, high-magnification image (iii), and the corresponding EDS mapping images of the electron-beam-degraded $CsPbBr_3$ seed NCs.

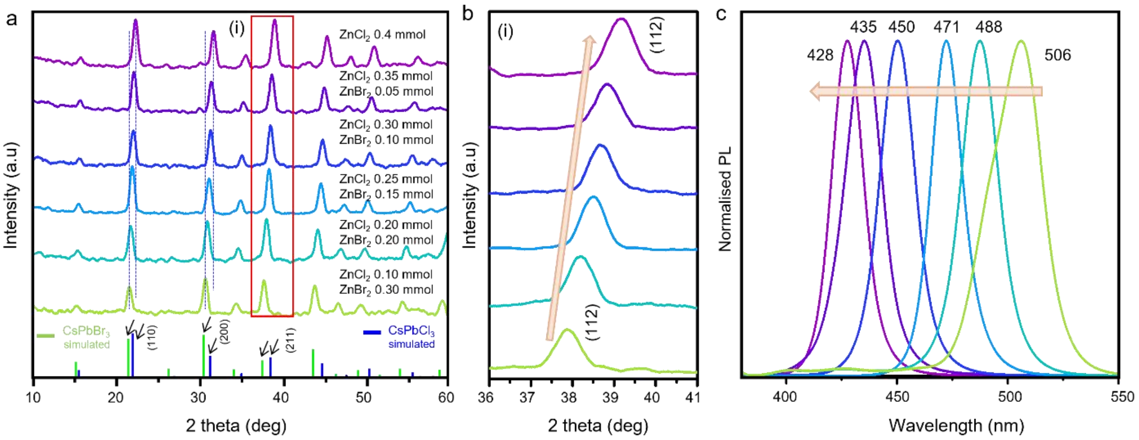

	ZnCl$_2$	ZnBr$_2$	Emission wavelength	
	0.40 mmol	-	428 nm	Deep blue
	0.35 mmol	0.05 mmol	435 nm	Deep blue
Main product	0.30 mmol	0.10 mmol	450 nm	Deep blue
	0.25 mmol	0.15 mmol	471 nm	Blue
	0.20 mmol	0.20 mmol	488 nm	Blue
	0.10 mmol	0.30 mmol	506 nm	

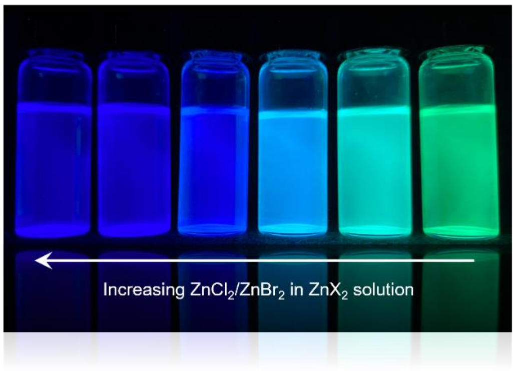

Figure S8. Modulation of the $CsPb(Br_{1-x}Cl_x)_3$ core halide compositions and bandgaps. a, PXRD patterns of the anion-exchanged and ZnS-encapsulated core/shell $CsPb(Br_{1-x}Cl_x)_3$/ZnS NC samples comprising different halide compositions; **b,** enlarged PXRD patterns between 36° and 41°; **c,** corresponding PL spectrum (λ_{ex} = 365 nm for all samples); **d,** typical quantities of $ZnCl_2$ and $ZnBr_2$ for six perovskite nanocrystal samples; **e,** digital photographs of perovskite colloidal solutions in hexane upon irradiation with UV lamp (λ_{ex} = 365 nm).

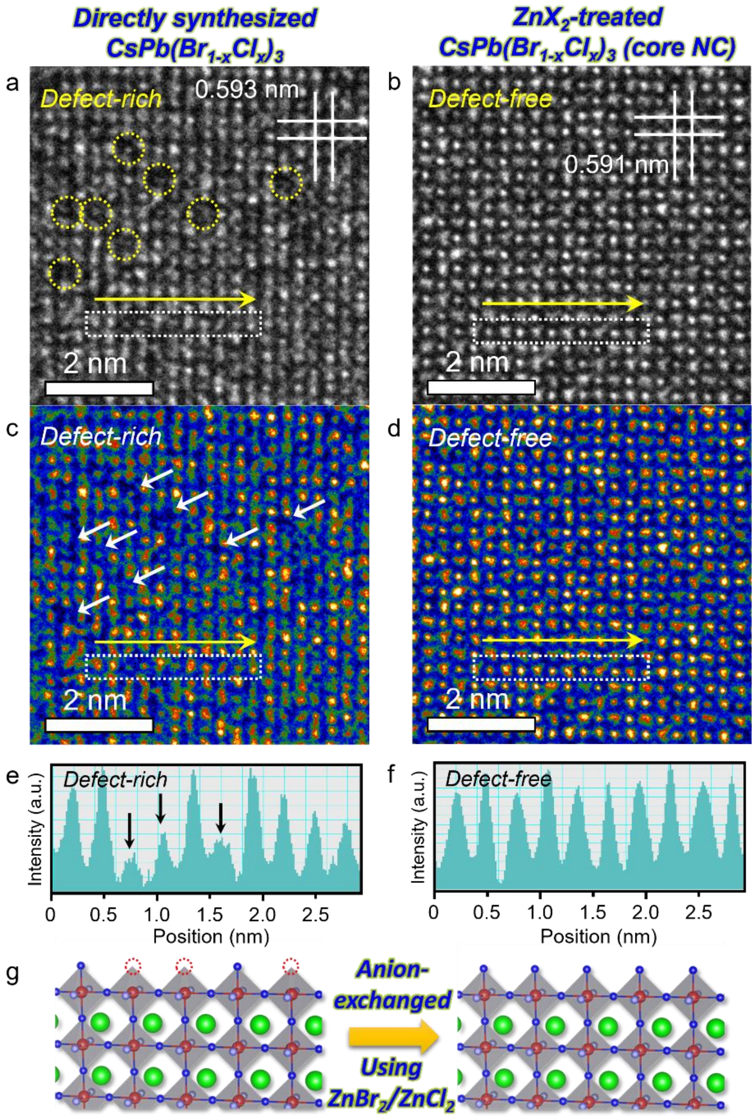

Figure S9. Defect densities in the $CsPb(Br_{1-x}Cl_x)_3$ NCs and core $CsPb(Br_{1-x}Cl_x)_3$ NCs directly synthesized from the $CsPbBr_3$ seed NCs after the ZnX_2 treatment. Atomic-resolution HRTEM images of **a,** directly synthesized $CsPb(Br_{1-x}Cl_x)_3$ NCs and **b,** $CsPb(Br_{1-x}Cl_x)_3$ core NCs, obtained after ZnX_2-treatment, respectively. Yellow dotted circles in (a) indicate defect sites on directly synthesized $CsPb(Br_{1-x}Cl_x)_3$ NCs. **c, d,** corresponding false-color images of HRTEM image of directly synthesized $CsPb(Br_{1-x}Cl_x)_3$ NCs and core NCs, respectively. White arrows in (c) and (d) indicate defect sites. **e, f,** Comparison of intensity profile analysis (white dotted boxes) in respective HRTEM image of directly synthesized $CsPb(Br_{1-x}Cl_x)_3$ NCs and core NCs further supports formation of defect sites in directly synthesized $CsPb(Br_{1-x}Cl_x)_3$ NCs. **g,** schematic of halide defective surface of directly synthesized $CsPb(Br_{1-x}Cl_x)_3$ NCs and defect passivated core NCs.

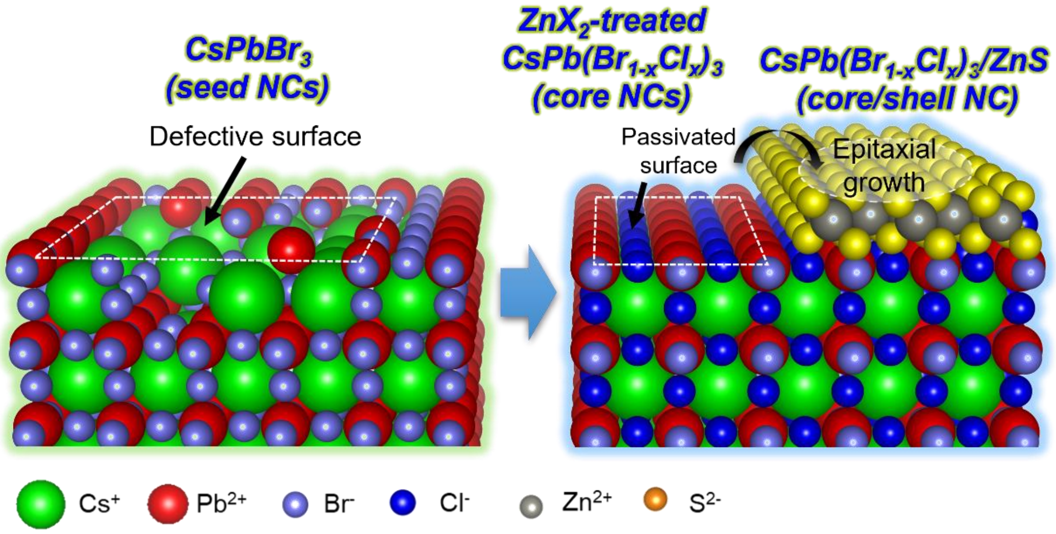

Figure S10. Schematic demonstrating surface evolution mechanism from $CsPbBr_3$ seed to $CsPb(Br_{1-x}Cl_x)_3$ core to core/shell $CsPb(Br_{1-x}Cl_x)_3/ZnS$ NCs.

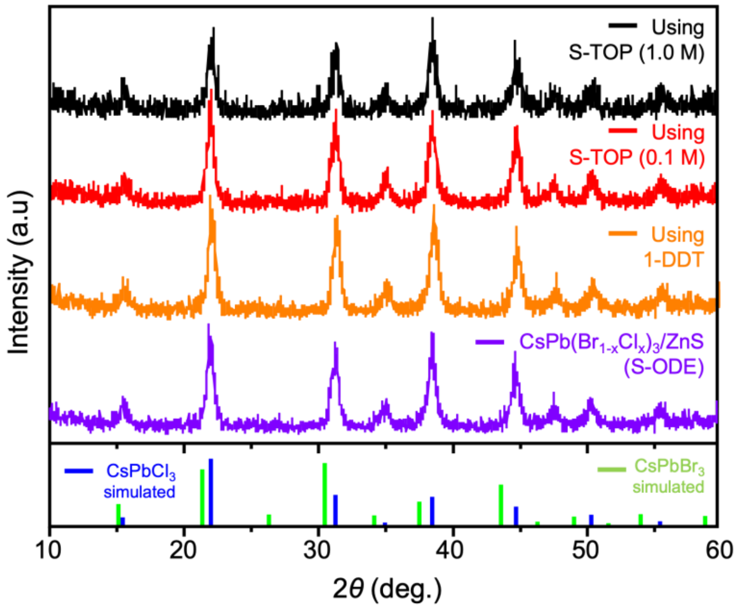

Figure S11. PXRD analysis of $CsPb(Br_{1-x}Cl_x)_3$ NCs obtained using less reactive sulfur precursor, such as S-TOP (1.0 M, 0.1 M), and 1-DDT.

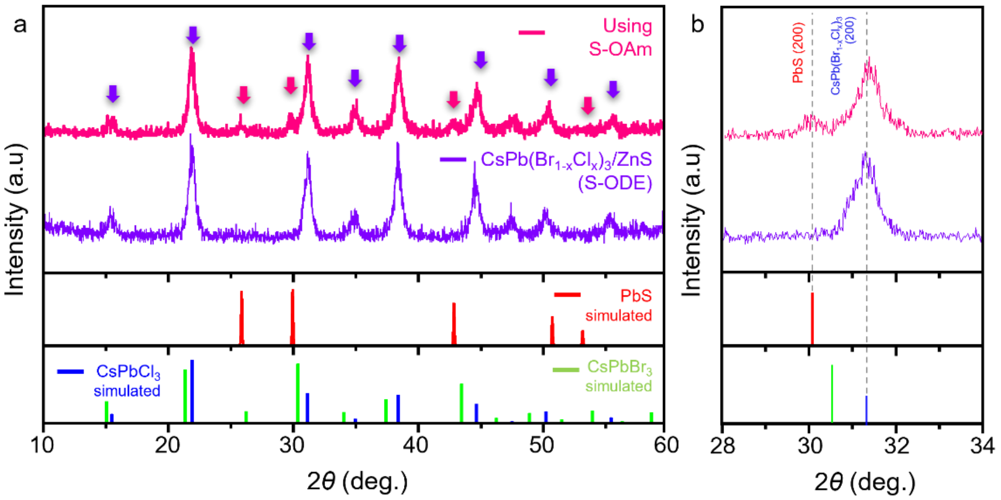

Figure S12. PXRD analysis of $CsPb(Br_{1-x}Cl_x)_3$ NCs obtained using S–OAm, demonstrating the formation of by-product PbS.

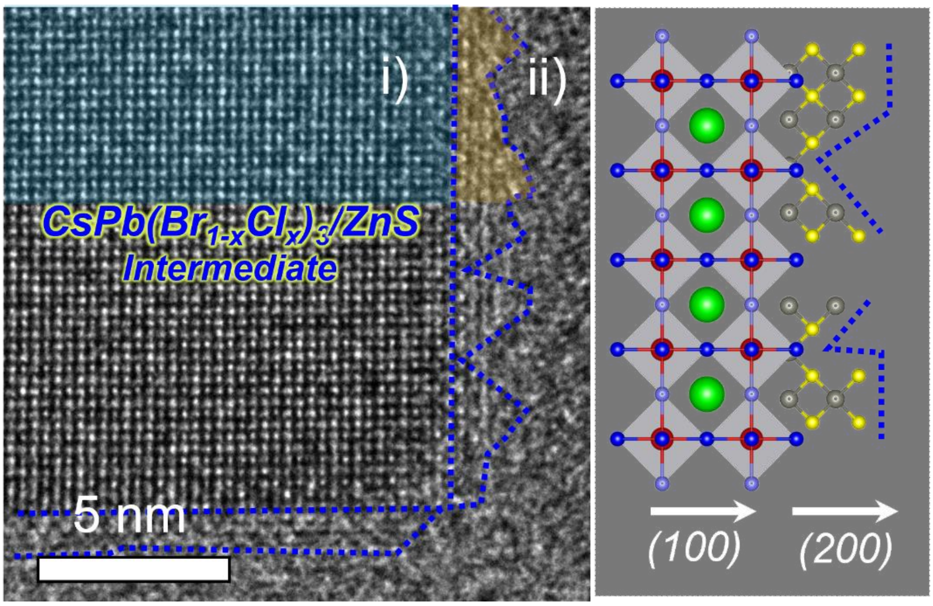

Figure S13. HRTEM characterization of core/shell $CsPb(Br_{1-x}Cl_x)_3$/ZnS intermediate (core/shell intermediate). A, HRTEM image and corresponding atomic model of core/shell intermediate, showing rough surface of partially grown ZnS shell (ii) on $CsPb(Br_{1-x}Cl_x)_3$ core (i) without temperature increasing step to 140 °C.

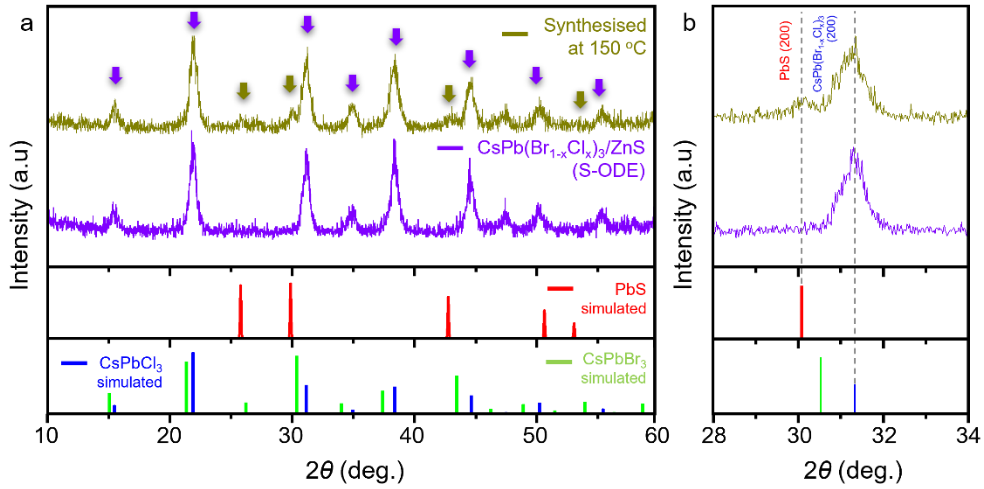

Figure S14. PXRD analysis of the $CsPb(Br_{1-x}Cl_x)_3$ NCs obtained when the encapsulation treatment was directly proceeded at 150 °C, demonstrating the formation of the by-product PbS.

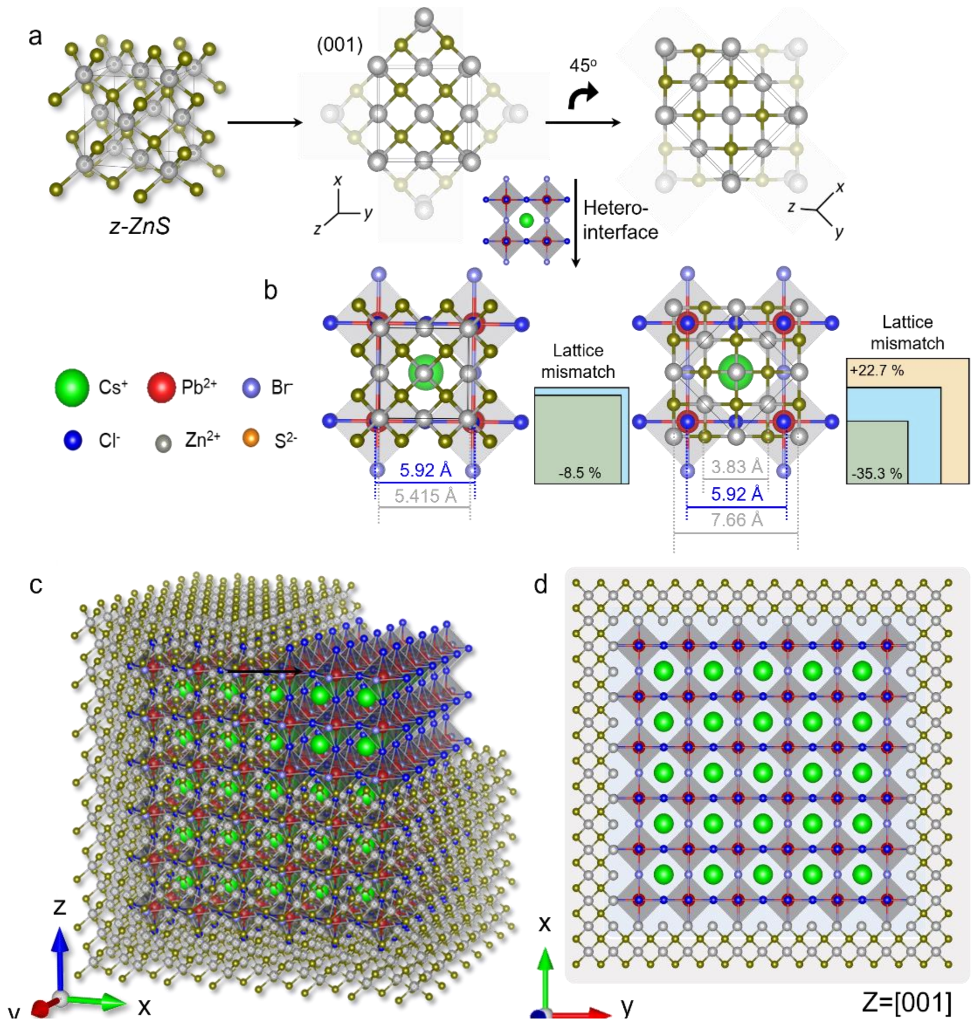

Figure S15. Calculation of the interfacial lattice mismatch between core $CsPb(Br_{1-x}Cl_x)_3$ NC and ZnS shell, and optimal core/shell crystal structure with epitaxial relation. a, Crystallographic projections highlighting cube-like zinc blende subunits within ZnS. The lattice parameters for core $CsPb(Br_{1-x}Cl_x)_3$ NC (5.920 Å) and z–ZnS shell (5.415 Å) for calculation were obtained by FFT analysis and theoretical value, respectively. **b,** Crystallographic projections of heterostructures of ZnS and core $CsPb(Br_{1-x}Cl_x)_3$ NC with corresponding lattice mismatch calculations. **c, d,** 3D and 2D-simulated atomic models of core/shell $CsPb(Br_{1-x}Cl_x)_3$/ZnS NCs.

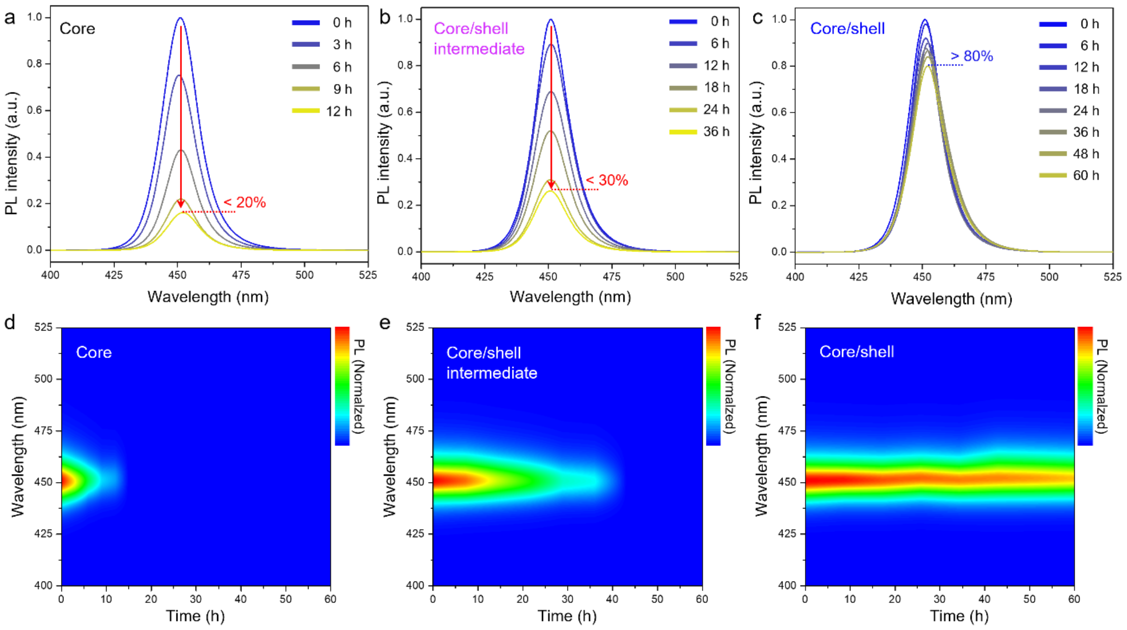

Figure. S16. Time-dependent PL spectra. a, d, core; **b, e,** core/shell intermediates, and **c, f,** core/shell NCs.

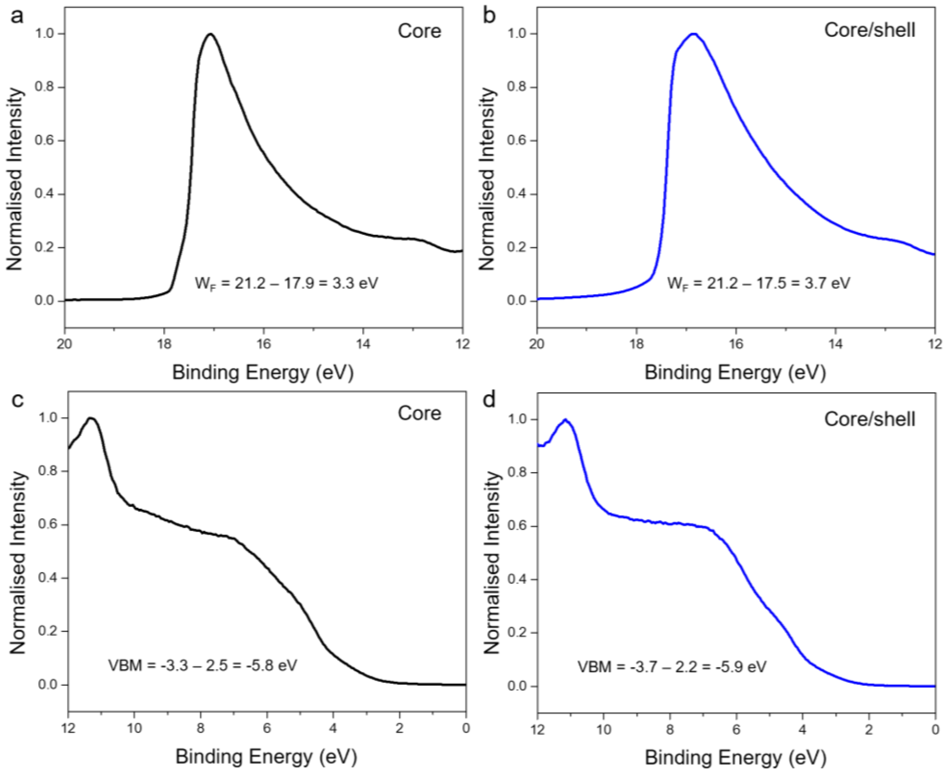

Figure S17. UPS characteristics. Second electron cut-off and valence band spectra of the **(a, c)** core and **(b, d)** core/shell NCs.

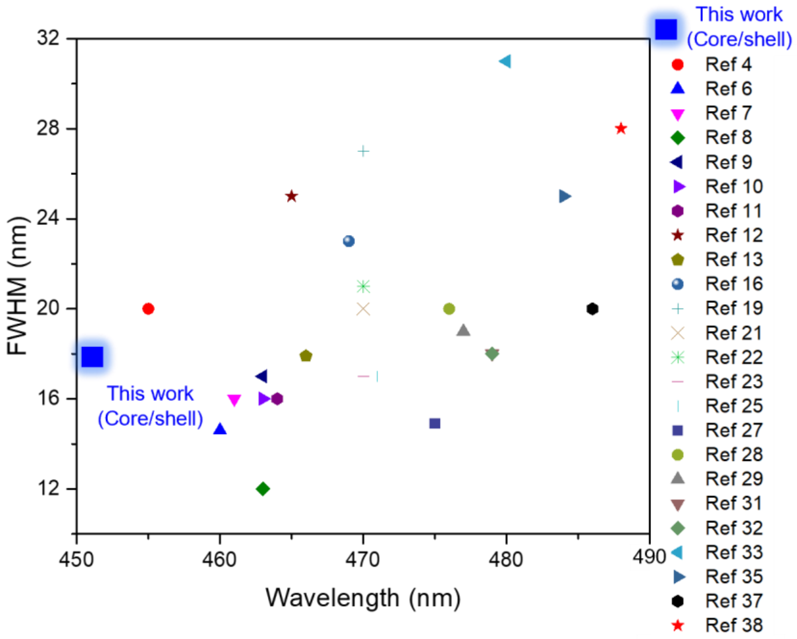

Figure S18. Comparison of the FWHM of the deep-blue core/shell PeLED fabricated in this study with those of other PeLEDs.

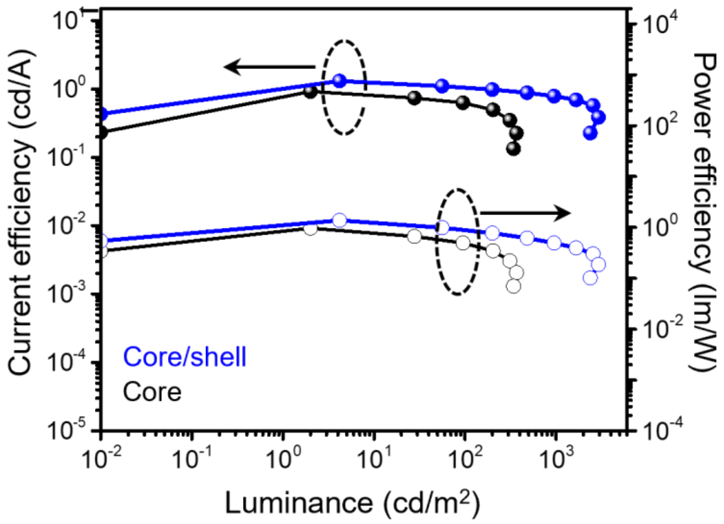

Figure S19. Luminance-dependent current efficiency (left axis) and power efficiency (right axis) of the core and core/shell PeLEDs.

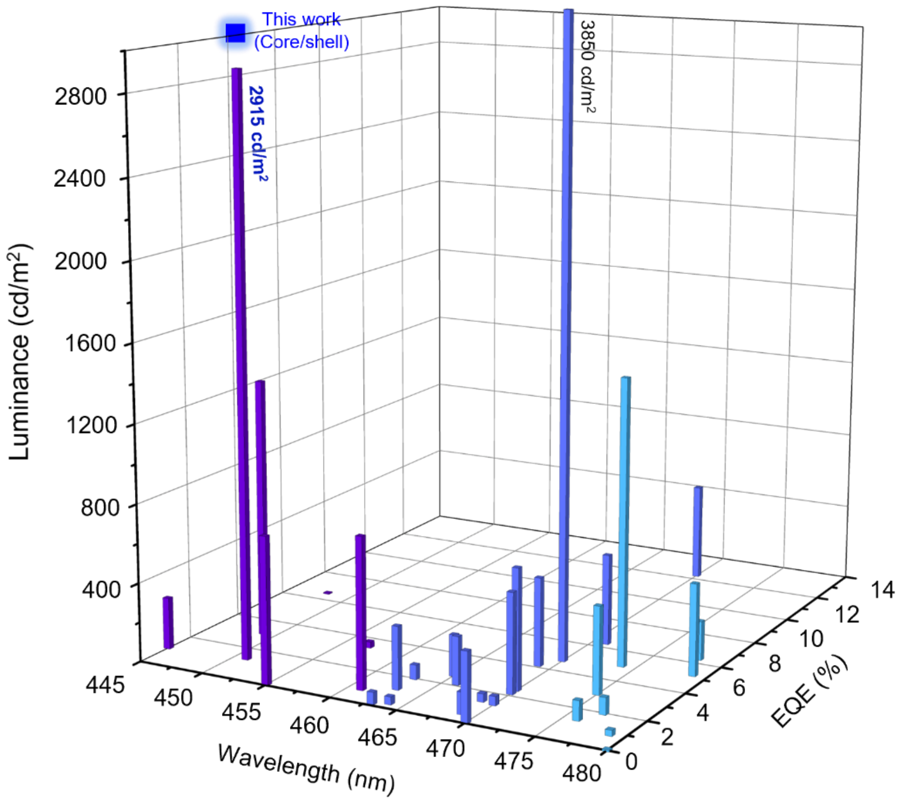

Figure S20. Comparison of the maximum EQE, luminance, and emission wavelength of the deep-blue core/shell PeLED fabricated in this study with those of other PeLEDs based on Table S3.

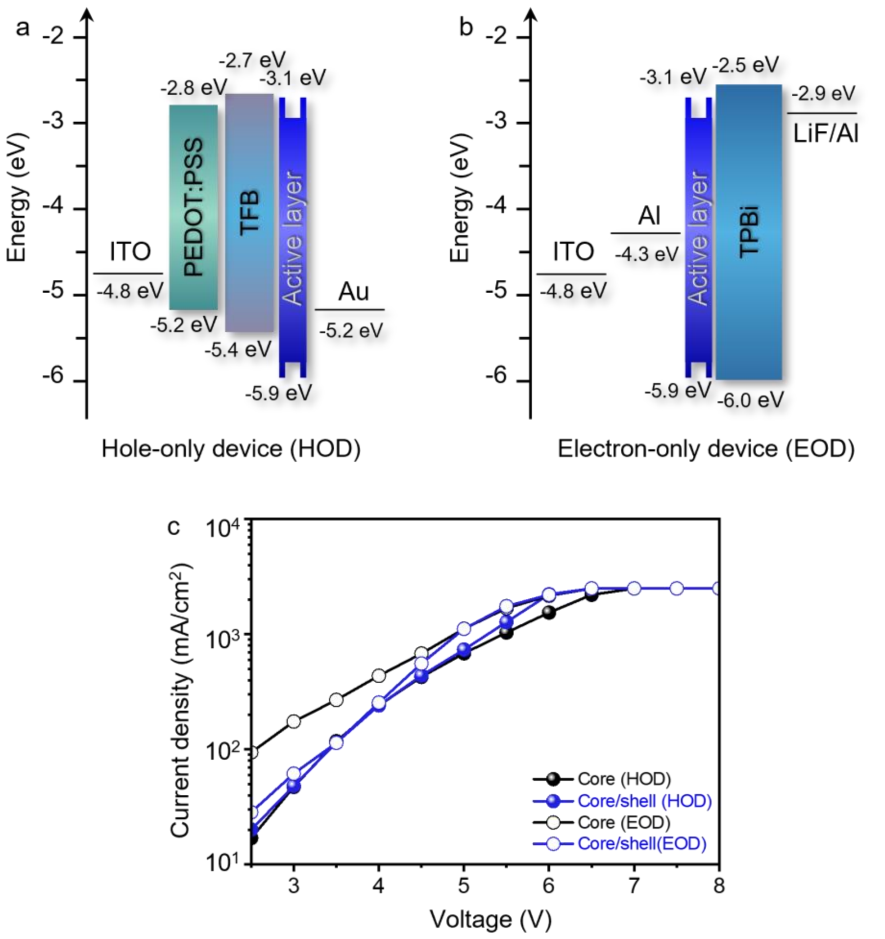

Figure S21. Schematic and energy-band diagrams. a, Hole-only device (HOD) and **b,** electron-only device (EOD). **c,** current density–voltage (*J*–*V*) characteristics of the HODs and EODs fabricated using the core and core/shell NCs, respectively.

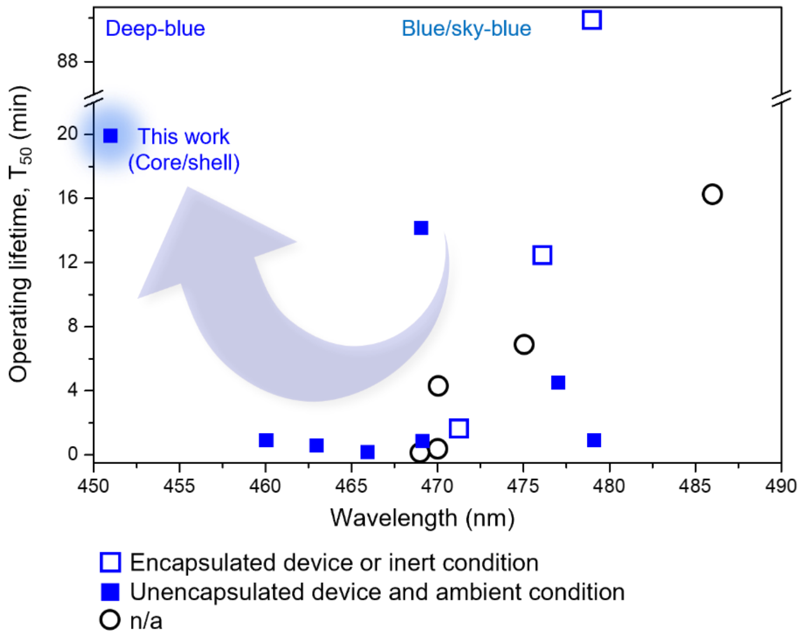

Figure S22. Comparison of the operating lifetimes (T_{50}) and emission wavelength of the deep-blue core/shell PeLEDs fabricated in this study with those of other blue PeLEDs with narrow FWHMs (< 25 nm) based on Table S3.

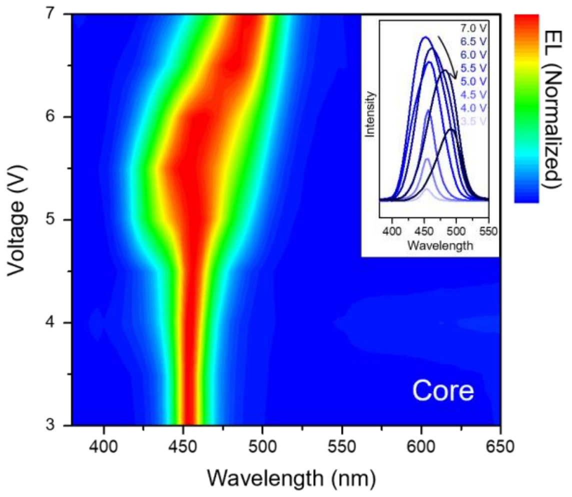

Figure S23. Driven-voltage-dependent electroluminescence spectra of the deep-blue core PeLEDs.

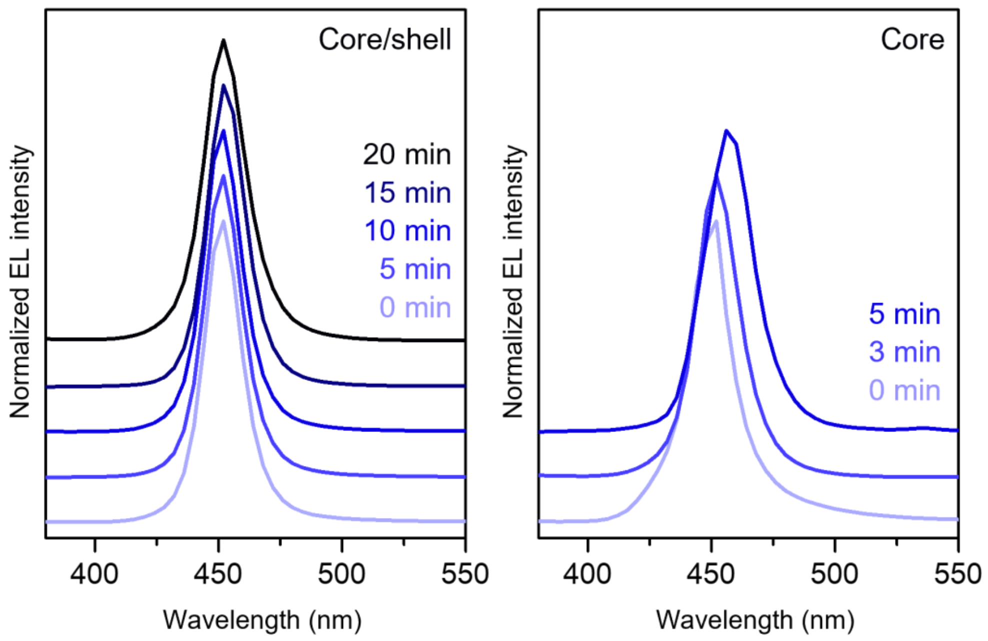

Figure S24. Electroluminescence spectra of the core and core/shell PeLEDs during the stability test.

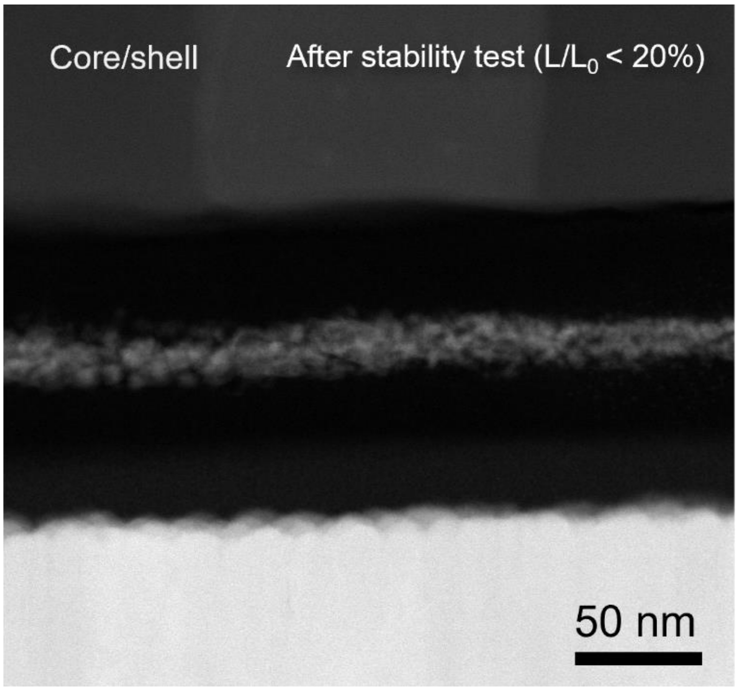

Figure S25. Cross-sectional HAADF–STEM image of the focused-ion-beam-milled core/shell $CsPb(Br_{1-x}Cl_x)_3$/ZnS-based PeLED devices after a severe stability test ($L/L_0 <$ 20%),

Table S1. TRPL spectroscopy parameters of the directly synthesized, core, and core/shell perovskites.

	Directly synthesized	Core	Core/shell
A_1	0.7874	0.6889	0.4809
τ_1	0.050 ns	0.070 ns	0.091 ns
A_2	0.1780	0.2568	0.3725
τ_2	0.82 ns	1.7 ns	1.9 ns
A_3	0.0294	0.0459	0.1207
τ_3	5.2 ns	8.5 ns	6.5 ns
A_4	0.0046	0.0072	0.0238
τ_4	26 ns	20 ns	16 ns
A_5	0.0006	0.0012	0.0021
τ_5	230 ns	190 ns	190 ns
τ_{avg}	0.60 ns	1.2 ns	2.3 ns

Table S2. Electrical performance of the core and core/shell-based deep-blue PeLEDs.

Sample	V_{on} [a] (V)	CE_{max} [b] (cd A^{-1})	PE_{max} [c] (lm W^{-1})	Luminance [d] (cd m^{-2})	EQE_{max} [e] (%)	λ_{EL} [f] (nm)	CIE [g] (x, y)
Core	2.94	0.92	0.96	366.2	0.83	451	(0.15, 0.03)
Core/shell	2.88	1.31	1.37	2916	1.32	451	(0.17, 0.06)

a) Turn-on voltage at 1 cd m^{-2}. b) Maximum CE. c) Maximum PE. d) Maximum luminance. e) Maximum EQE. h) Electroluminescence peak wavelength.
g) CIE 1931 color coordinates.

Table S3. Comparison of the state-of-the-art mixed-halide blue PeLEDs reported in previous studies with the deep-blue PeLED fabricated in this study.

Perovskite emission layer	Wave length (nm)	EQE_{max} (%)	Luminance (cd m^{-2})	FWHM (nm)	Operational lifetime (T_{50})	Device structure	Device measurement conditions	Year [Ref.]
**$CsPb(Br_{1-x}Cl_x)_3$/ZnS**	**451**	**1.32**	**2916**	**17.8**	**1192 s**	**ITO/PEDOT:PSS/TFB/core/shell perovskite/TPBi/LiF/Al**	**Unencapsulated/ ambient condition**	**This work**
$Cs_3Cu_2I_5$	445	1.12	262.6	-	108 h @ 6.7 V	ITO/p-NiO/perovskite/TPBi/LiF/Al	At pressure below 2.0 x 10^{-4} Pa	2020[1]
$CsEuBr_3$	448	6.5	5.2	-	50 min @ 15.9 cd m^{-2}	ITO/LiF/perovskite/LiF/TPBi/LiF/Al	At N_2-filled glovebox	2021[2]
BI_2PbBr_4	449	3.08	1315	-	3.5 h @ 5 V	ITO/PEDOT:PSS/Poly-TPD/perovskite/TPBi/LiF/Al	Encapsulated (epoxy)	2021[3]
$CsPb(Br/Cl)_3$	455	0.07	742	20	-	ITO/PEDOT:PSS/PVK/perovskite/TPBi/LiF/Al	-	2015[4]
$CsPbBr_{2.5}Cl_{0.5}$+GA10	457	3.41	32	-	6.5 min	ITO/PVK/perovskite/TPBi/LiF/Al	Encapsulated/ambient condition	2022[5]
Ni^{2+}-doped $CsPb(Br_{1.7}Cl_{1.3})_3$	460	1.35	33	14.6	51.5 s @ 3.7 V	ITO/PEDOT:PSS/poly-TPD/perovskite/TPBi/LiF/Al	Ambient condition	2019[6]
$CsPbBr_{1.3}Cl_{1.7}$	461	0.8	763	16	-	ITO/PEDOT:PSS/poly-TPD/perovskite/TPBi/LiF/Al	-	2019[7]
$CsPbBr_3$	463	0.12	62	12	-	ITO/PEDOT:PSS/Poly-TPD/perovskite/TPBi/LiF/Al	-	2018[8]
$CsPbBr_3$	463	2	74	17	-	ITO/PEDOT:PSS/PTAA/PEABr/ /perovskite/TPBi/LiF/Al	-	2022[9]

$CsPb(Br/Cl)_3$	463	1.2	318	16	~30 s @ 5.0 V	ITO/PEDOT:PSS/poly-TPD/CBP/perovskite/B3PYMPM/LiF/Al	Ambient condition	2019[10]
$CsPbBr_3$	464	0.3	40	16	-	ITO/PEDOT:PSS/PD/TFB/perovskite/TPBi/Ca/Ag	-	2019[11]
P-PDABr_2:PEABr:CsBr:PbBr	465	2.6	211	25	13.5 min @ 0.35 mA m^{-2}	ITO/PVK/PFI/perovskite/3TPYMB/Liq/Al	Ambient condition	2019[12]
$CsMn_yPb_{1-y}Br_xCl_{3-x}$	466	2.12	245	17.9	12 s @ 1 mA cm^{-2}	ITO/PEDOT:PSS/TFB/PFI/perovskite/TPBi/LiF/Al	Unencapsulated/ambient condition	2018[13]
(Cs/FA/p-F-PEA)Pb(Cl/Br)$_3$	469	4.14	451	-	14 min @ 1 mA cm^{-2}	ITO/PEDOT:PSS/perovskite/TPBi/LiF/Al	Encapsulated (glass cap)/at glovebox	2021[14]
$CsPbBr_3$	469	1.42	41.8	-	42 s @ 1 mA cm^{-2}	ITO/PEDOT:PSS/Poly-TPD/perovskite/TPBi/LiF/Al	Unencapsulated/ambient condition	2019[15]
$CsPbBr_xCl_{3-x}$	469	0.5	111	23	1 s @ 10 mA cm^{-2}	ITO/PEDOT:PSS/TFB/PFI/perovskite/TPBi/LiF/Al	-	2018[16]
$CsPb(Br_{1-x}Cl_x)_3$:Ni	470	2.4	612	-	-	glass/ITO/PEDOT:PSS/TFB/PFI/perovskite/TPBi/LiF/Al	-	2021[17]
$CsPbBr_3$	470	12.3	~500	-	20 min @ 90 cd m^{-2}	ITO/PEDOT:PSS/PTAA/perovskite/TPBi/LiF/Al	Encapsulated (epoxy)/ambient condition	2020[18]
$CsPbBr_3$	470	4.7	3850	27	12 h @ 102 cd m^{-2}	ITO/PEDOT:PSS//PVK/perovskite/ZnO/Ag	Unencapsulated/ambient condition	2021[19]
Ni^{2+}-doped $CsPbBr_xCl_{3-x}$	470	2.4	612	-	-	ITO/PEDOT:PSS/TFB/PFI/perovskite/TPBi/LiF/Al	-	2020[20]
$CsPbBr_xCl_{3-x}$	470	0.07	350	20	Spectra shifted at ~15 nm	ITO/NiOx/perovskite/TPBi/LiF/Al	-	2017[21]
PEA-$CsPbCl_3$	470	2.15	507	21	23.6 s @ 7 V	ITO/PEDOT:PSS/Poly-TPD/PVK/perovskite/TmPyPB/LiF/Al	-	2020[22]
$CsPbBr_xCl_{3-x}$	470	1.34	46.7	17	257 s @ 3.5 V	ITO/PEDOT:PSS/Poly-TPD/PFN-Cl/perovskite/TPBi/LiF/Al	-	2020[23]
$CsPbBr_xCl_{3-x}$	471	>2.5	420	-	1.1 h @ 1 mA m^{-2} 200 K	ITO/PEDOT:PSS/TFB:PMMA/PFI/perovskite/PO-T2T/Alq3/Al	Encapsulated/ambient atmosphere	2022[24]
$CsPb(Br_xCl_{1-x})_3$	471	6.3	465	17	99 s @ 4.5 V	ITO/TFB/PFI/perovskites/3TPYMB/Liq/Al	Encapsulated/ambient condition	2020[25]
FA-$CsPb(Cl_{0.5}Br_{0.5})_3$	474	5.01	1452	-	1056 s (L_0 = 100 cd m^{-2})	ITO/PEDOT:PSS/Poly-TPD/perovskite/TPBi/LiF/Al	-	2022[26]
DPPACl:$CsPbBr_2Cl$	475	3.03	442	14.9	7 min @ 5 V	ITO/PEDOT:PSS/TFB/perovskite/PMMA/TPBi/LiF/Al	-	2020[27]
PEA2$(Rb_xCs_{1-x})_2Pb_3Br_1$	476	1.35	100.6	20	14.5 min @ 4.5 V (L_0= 15 cd m^{-2})	ITO/PEDOT:PSS/perovskite/TmPyPB/LiF/Al	At N_2-filled glovebox	2019[28]
$CsPb(Br/Cl)_3$	477	1.96	86.95	19	4.5 min @ 4.0 V	ITO/PEDOT:PSS/poly-TPD/PVK/perovskite/POT2T/LiF/Al	Unencapsulated/ambient condition	2020[29]
GABA-$CsPbBr_3$	478	6.3	~200	-	2.5 min (L_0= 200 cd m^{-2})	ITO/PEDOT:PSS:PFI/PVK/Perovskite/TPBi/LiF/Al	Encapsulated (resin)/ambient atmosphere	2020[30]
PEA2$Cs_{1.6}MA_{0.4}Pb_3Br_{1-x}Cl_x$-DPPOC	479	5.2	468	18	90 min (L_0= 100 cd m^{-2})	ITO/PEDOT:PSS:PFI/Perovskite/TPBi/LiF/Al	Encapsulated (resin)/ambient atmosphere	2020[31]
$CsPbBr_xCl_{3-x}$	479	0.86	29.9	18	50 s @ 4.0 V	ITO/PEDOT:PSS/Poly-TPD/perovskite/TPBi/LiF/Al	Ambient condition	2019[32]
$CsPb(Br_{1-x}Cl_x)_3$	480	0.0074	8.7	31	spectra shift @ ~10 s	ITO/ZnO/perovskites/TFB/MoO_3/Al	Encapsulated (epoxy)	2016[33]
$CsxFA_{1-x}PbBr_3$	483	9.5	700	-	250 s (L_0= 100 cd m^{-2})	ITO/NiOx/TFB/PVK/perovskite/TPBi/LiF/Al	At N_2-filled glovebox	2019[34]
$CsPb(Br_{1-x}Cl_x)_3$	484	2.01	4015	25	300 min @ 25 mA cm^{-2}	ITO/LiF/perovskite/LiF/Bphen/LiF/Al	-	2020[35]
$CsPbBr_3$:PEACl:YCl3	485	11	9040	-	100 min @ 3.2 V (L_0= 100 cd m^2)	ITO/PEDOT:PSS/perovskite/TPBi/LiF/Al	At N_2-filled glovebox	2019[36]
$CsPbBr_{3-x}Cl_x$	486	12.8	1390	20	16.2 min @ 150 cd m^{-2}	ITO/ETA-PEDOT:PSS/perovskite/TPBi/LiF/Al	-	2021[37]
PEA-$Cs_{1-x}DA_xPbBr_{2.3}Cl_{0.7}$	488	14.71	5015	28	900 s @ 0.2 mA cm^{-2}	ITO/PEDOT:PSS/perovskite/TOPO/TPBi/LiF/Al	-	2022[38]
$CsPb(Br_{0.84}Cl_{0.16})_3$	489	10.3	~400	-	390 s (L_0 = 100 cd m^{-2})	ITO/PVK/perovksite/TPBi/LiF/Al	At N_2	2022[39]

Table S4. Variation of CIE coordinates of core/shell based PeLED according to the applied voltage.

Sample	Voltage (V)								
	3.0 V	3.5 V	4.0 V	4.5 V	5.0 V	5.5 V	6.0 V	6.5 V	7.0 V
Core/shell CIE (x, y)	(0.170, 0.060)	(0.170, 0.061)	(0.170, 0.062)	(0.170, 0.062)	(0.171, 0.063)	(0.171, 0.067)	(0.172, 0.071)	(0.174, 0.075)	(0.176, 0.079)

References

[1] L. Wang, Z. Shi, Z. Ma, D. Yang, F. Zhang, X. Ji, M. Wang, X. Chen, G. Na, S. Chen, D. Wu, Y. Zhang, X. Li, L. Zhang, C. Shan, *Nano Lett.* **2020**, *20*, 3568.
[2] J. Luo, L. Yang, Z. Tan, W. Xie, Q. Sun, J. Li, P. Du, Q. Xiao, L. Wang, X. Zhao, G. Niu, L. Gao, S. Jin, J. Tang, *Adv. Mater.* **2021**, *33*, 2101903.
[3] S. Yan, W. Tian, H. Chen, K. Tang, T. Lin, G. Zhong, L. Qiu, X. Pan, W. Wang, *Adv. Opt. Mater.* **2021**, *9*, 2001709.
[4] J. Song, J. Li, X. Li, L. Xu, Y. Dong, H. Zeng, *Adv. Mater.* **2015**, *27*, 7162.
[5] Y.-H. Zhou, C. Wang, S. Yuan, C. Zou, Z. Su, K.-l. Wang, Y. Xia, B. Wang, D. Di, Z.-K. Wang, L.-S. Liao, *J. Am. Chem. Soc.* **2022**, *144*, 18470.
[6] B.-B. Zhang, S. Yuan, J.-P. Ma, Y. Zhou, J. Hou, X. Chen, W. Zheng, H. Shen, X.-C. Wang, B. Sun, O. M. Bakr, L.-S. Liao, H.-T. Sun, *J. Am. Chem. Soc.* **2019**, *141*, 15423.
[7] D. Yang, X. Li, Y. Wu, C. Wei, Z. Qin, C. Zhang, Z. Sun, Y. Li, Y. Wang, H. Zeng, *Adv. Opt. Mater.* **2019**, *7*, 1900276.
[8] Y. Wu, C. Wei, X. Li, Y. Li, S. Qiu, W. Shen, B. Cai, Z. Sun, D. Yang, Z. Deng, H. Zeng, *ACS Energy Lett.* **2018**, *3*, 2030.
[9] H. Wang, F. Ye, J. Sun, Z. Wang, C. Zhang, J. Qian, X. Zhang, W. C. H. Choy, X. W. Sun, K. Wang, W. Zhao, *ACS Energy Lett.* **2022**, *7*, 1137.
[10] S. T. Ochsenbein, F. Krieg, Y. Shynkarenko, G. Rainò, M. V. Kovalenko, *ACS Appl. Mater. Interfaces* **2019**, *11*, 21655.
[11] R. L. Z. Hoye, M.-L. Lai, M. Anaya, Y. Tong, K. Gałkowski, T. Doherty, W. Li, T. N. Huq, S. Mackowski, L. Polavarapu, J. Feldmann, J. L. MacManus-Driscoll, R. H. Friend, A. S. Urban, S. D. Stranks, *ACS Energy Lett.* **2019**, *4*, 1181.
[12] S. Yuan, Z.-K. Wang, L.-X. Xiao, C.-F. Zhang, S.-Y. Yang, B.-B. Chen, H.-T. Ge, Q.-S. Tian, Y. Jin, L.-S. Liao, *Adv. Mater.* **2019**, *31*, 1904319.
[13] S. Hou, M. K. Gangishetty, Q. Quan, D. N. Congreve, *Joule* **2018**, *2*, 2421.
[14] Y. Shen, K.-C. Shen, Y.-Q. Li, M. Guo, J. Wang, Y. Ye, F.-M. Xie, H. Ren, X. Gao, F. Song, J.-X. Tang, *Adv. Funct. Mater.* **2021**, *31*, 2006736.
[15] C. Zhang, Q. Wan, B. Wang, W. Zheng, M. Liu, Q. Zhang, L. Kong, L. Li, *J. Phys. Chem. C.* **2019**, *123*, 26161.
[16] M. K. Gangishetty, S. Hou, Q. Quan, D. N. Congreve, *Adv. Mater.* **2018**, *30*, 1706226.
[17] P. Wang, Z. Wu, M. Wu, J. Wei, Y. Sun, Z. Zhao, *J. Mater. Sci.* **2021**, *56*, 4161.
[18] Y. Dong, Y.-K. Wang, F. Yuan, A. Johnston, Y. Liu, D. Ma, M.-J. Choi, B. Chen, M. Chekini, S.-W. Baek, L. K. Sagar, J. Fan, Y. Hou, M. Wu, S. Lee, B. Sun, S. Hoogland, R. Quintero-Bermudez, H. Ebe, P. Todorovic, F. Dinic, P. Li, H. T. Kung, M. I. Saidaminov, E. Kumacheva, E. Spiecker, L.-S. Liao, O. Voznyy, Z.-H. Lu, E. H. Sargent, *Nat. Nanotechnol.* **2020**, *15*, 668.
[19] C. Bi, Z. Yao, X. Sun, X. Wei, J. Wang, J. Tian, *Adv. Mater.* **2021**, *33*, 2006722.

[20] G. Pan, X. Bai, W. Xu, X. Chen, Y. Zhai, J. Zhu, H. Shao, N. Ding, L. Xu, B. Dong, Y. Mao, H. Song, *ACS Appl. Mater. Interfaces* **2020**, *12*, 14195.
[21] E.-P. Yao, Z. Yang, L. Meng, P. Sun, S. Dong, Y. Yang, Y. Yang, *Adv. Mater.* **2017**, *29*, 1606859.
[22] H. Shao, Y. Zhai, X. Wu, W. Xu, L. Xu, B. Dong, X. Bai, H. Cui, H. Song, *Nanoscale* **2020**, *12*, 11728.
[23] Y. S. Shin, Y. J. Yoon, J. Heo, S. Song, J. W. Kim, S. Y. Park, H. W. Cho, G.-H. Kim, J. Y. Kim, *ACS Appl. Mater. Interfaces* **2020**, *12*, 35740.
[24] S. Yuan, X. Zheng, W.-S. Shen, J. Liu, L.-S. Cui, C. Zhang, Q.-S. Tian, J.-J. Wu, Y.-H. Zhou, X.-D. Wang, Z.-K. Wang, P. Han, J. M. Luther, O. M. Bakr, L.-S. Liao, *ACS Energy Lett.* **2022**, *7*, 1348.
[25] X. Zheng, S. Yuan, J. Liu, J. Yin, F. Yuan, W.-S. Shen, K. Yao, M. Wei, C. Zhou, K. Song, B.-B. Zhang, Y. Lin, M. N. Hedhili, N. Wehbe, Y. Han, H.-T. Sun, Z.-H. Lu, T. D. Anthopoulos, O. F. Mohammed, E. H. Sargent, L.-S. Liao, O. M. Bakr, *ACS Energy Lett.* **2020**, *5*, 793.
[26] L. Gao, Y. Zhang, L. Gou, Q. Wang, M. Wang, W. Zheng, Y. Wang, H.-L. Yip, J. Zhang, *Light Sci. Appl.* **2022**, *11*, 346.
[27] Y. C. Kim, H. J. An, D. H. Kim, J.-M. Myoung, Y. J. Heo, J. H. Cho, *Adv. Funct. Mater.* **2021**, *31*, 2005553.
[28] Y. Jiang, C. Qin, M. Cui, T. He, K. Liu, Y. Huang, M. Luo, L. Zhang, H. Xu, S. Li, J. Wei, Z. Liu, H. Wang, G.-H. Kim, M. Yuan, J. Chen, *Nat. Commun.* **2019**, *10*, 1868.
[29] F. Yang, H. Chen, R. Zhang, X. Liu, W. Zhang, J. Zhang, F. Gao, L. Wang, *Adv. Funct. Mater.* **2020**, *30*, 1908760.
[30] Y.-K. Wang, D. Ma, F. Yuan, K. Singh, J. M. Pina, A. Johnston, Y. Dong, C. Zhou, B. Chen, B. Sun, H. Ebe, J. Fan, M.-J. Sun, Y. Gao, Z.-H. Lu, O. Voznyy, L.-S. Liao, E. H. Sargent, *Nat. Commun.* **2020**, *11*, 3674.
[31] D. Ma, P. Todorović, S. Meshkat, M. I. Saidaminov, Y.-K. Wang, B. Chen, P. Li, B. Scheffel, R. Quintero-Bermudez, J. Z. Fan, Y. Dong, B. Sun, C. Xu, C. Zhou, Y. Hou, X. Li, Y. Kang, O. Voznyy, Z.-H. Lu, D. Ban, E. H. Sargent, *J. Am. Chem. Soc.* **2020**, *142*, 5126.
[32] Y. S. Shin, Y. J. Yoon, K. T. Lee, J. Jeong, S. Y. Park, G.-H. Kim, J. Y. Kim, *ACS Appl. Mater. Interfaces* **2019**, *11*, 23401.
[33] G. Li, F. W. R. Rivarola, N. J. L. K. Davis, S. Bai, T. C. Jellicoe, F. de la Peña, S. Hou, C. Ducati, F. Gao, R. H. Friend, N. C. Greenham, Z.-K. Tan, *Adv. Mater.* **2016**, *28*, 3528.
[34] Y. Liu, J. Cui, K. Du, H. Tian, Z. He, Q. Zhou, Z. Yang, Y. Deng, D. Chen, X. Zuo, Y. Ren, L. Wang, H. Zhu, B. Zhao, D. Di, J. Wang, R. H. Friend, Y. Jin, *Nat. Photonics* **2019**, *13*, 760.
[35] F. Yuan, C. Ran, L. Zhang, H. Dong, B. Jiao, X. Hou, J. Li, Z. Wu, *ACS Energy Lett.* **2020**, *5*, 1062.
[36] Q. Wang, X. Wang, Z. Yang, N. Zhou, Y. Deng, J. Zhao, X. Xiao, P. Rudd, A. Moran, Y. Yan, J. Huang, *Nat. Commun.* **2019**, *10*, 5633.
[37] Y. Shen, H.-Y. Wu, Y.-Q. Li, K.-C. Shen, X. Gao, F. Song, J.-X. Tang, *Adv. Funct. Mater.* **2021**, *31*, 2103870.
[38] B. Liu, J. Li, G. Wang, F. Ye, H. Yan, M. Zhang, S.-C. Dong, L. Lu, P. Huang, T. He, P. Xu, H.-S. Kwok, G. Li, *Sci. Adv.* **2022**, *8*, eabq0138.
[39] S. Chu, Y. Zhang, P. Xiao, W. Chen, R. Tang, Y. Shao, T. Chen, X. Zhang, F. Liu, Z. Xiao, *Adv. Mater.* **2022**, *34*, 2108939.
